# The deregulation of miR-17/CCND1 axis during neuroendocrine transdifferentiation of LNCaP prostate cancer cells

**DOI:** 10.1371/journal.pone.0200472

**Published:** 2018-07-12

**Authors:** Jaroslaw Thomas Dankert, Marc Wiesehöfer, Elena Dilara Czyrnik, Bernhard B. Singer, Nicola von Ostau, Gunther Wennemuth

**Affiliations:** Institute of Anatomy, University Hospital, Duisburg-Essen University, Essen, Germany; University of Leeds, Faculty of Medicine and Health, UNITED KINGDOM

## Abstract

Prostate carcinoma contain foci of neuroendocrine transdifferentiation, resulting in an increase of androgen-independent neuroendocrine-like (NE) tumor cells, whose number significantly correlates with tumor aggressiveness and thus lower survival rate. Neuroendocrine transdifferentiation of prostate cancer cells and a potential role of miRNAs within this process are poorly understood. MicroRNAs are small non-coding RNAs which post-transcriptionally regulate gene expression. The aim of this project was to identify new genes and miRNAs involved in neuroendocrine transdifferentiation. LNCaP prostate cancer cells were differentiated to NE-like cancer cells and microarray analyses were performed. Microarray results have been validated for the eight most deregulated mRNAs and microRNAs via qRT-PCR and analyzed with different algorithms to predict new targets for deregulated microRNAs. The induced CyclinD1 gene could be validated as new target gene for the repressed miR-17 family containing miR-17, miR-20a, miR-20b, miR-106a and miR-106b via reporter gene assays and Western Blot. Functional analysis of miR-17 family shows a high influence on cell proliferation, colony forming ability and apoptosis in LNCaP cells. Our data demonstrate wide changes in mRNA and microRNA expression during neuroendocrine transdifferentiation of LNCaP cells and confirm new mRNA-miRNA interactions with potential roles in NE-transdifferentiation of prostate carcinoma.

## Introduction

Prostate cancer (PCa) is the second most common diagnosed cancer type in male worldwide contributing 15% of the total number of new cancer cases diagnosed. Furthermore, two thirds of cases of prostate cancer are diagnosed in the western world and lead to a major health problem in many industrialized countries [1]. Androgens are one critical factor for the development and progression of prostate tumors and are the main therapeutic target consisting of androgen depletion or androgen receptor (AR) blocking in advanced and metastatic prostate cancer disease. However, most patients relapse and develop androgen-independent and more aggressive forms of prostate cancer without promising cure options [2]. There are several mechanisms discussed which can lead to the switch from androgen dependent to independent tumor growth including AR overexpression, AR mutation or AR bypass through activation of alternative growth pathways. Furthermore, androgen deprivation therapy induces neuroendocrine transdifferentiation (NETD) of prostate cancer cells to neuroendocrine- (NE-) like tumor cells (NETC) [3].

NE cells in healthy prostate are part of the epithelial compartment and are thought to be involved in the regulation, secretion, differentiation and proliferation of prostatic epithelium. These functions are based on their secretion of diverse neurosecretory products, such as chromogranin A and B, serotonin, thyroid-stimulating hormone-like peptide, bombesin or somatostatin. Furthermore, NE cells are post-mitotic and terminally differentiated, lacking AR and Ki67 expression [4]. Prostatic NETC share these NE cell characteristics which result in resistance of NE cell populations in prostatic adenocarcinoma against androgen deprivation therapy and castration [5]. NETC are located in foci inside differentiated prostate cancer, in contrast to small cell carcinoma being usually entirely composed of NE tumor cells. NETD is increased in high-grade and high-stage prostatic tumors promoting androgen-independent growth and tumorigenesis as well as invasion and metastasis of prostate cancer cells [6]. Furthermore, several studies suggested a correlation of NETD markers detected in sera or via immunohistochemistry and Gleason grade and thus poor clinical outcome [7]. Although NETD in prostate adenocarcinoma received increased attention in recent years, the mechanisms leading to NETD and the exact role of NETC in disease progression are not fully understood yet. Several *in vitro* approaches exist to induce NETD of prostate cancer cells including androgen depletion, treatment with cytokines (i.e. IL-6), cAMP or Forskolin [8]. It is assumed that microRNAs (miRNAs) may play a role in NETD as the deregulation of miRNAs is constantly reported as one important mechanism that accounts to development and progression of different tumor entities [9].

MiRNAs are endogenous short non-coding RNA molecules that post-transcriptionally regulate protein expression by preferentially binding to specific sequences in the 3’-untranslated region (3’UTR) of mRNA targets. This interaction facilitated by the RISC (RNA-induced silencing) complex containing Argonaute (Ago-) proteins results in inhibition of protein synthesis, either by translational repression or degradation of the corresponding target mRNA. MiRNAs can function both as tumor suppressors and as oncogenes depending on their controlled target gene in the appropriate tissue.

The deregulation of miRNAs in prostate cancer has been studied extensively [10]. We had previously described the miRNA expression profile of prostate cancer at different stages of malignancy and identified novel targets for deregulated miRNAs in prostate cancer [11–15]. However, only few studies investigated the impact and function of single miRNAs related to neuroendocrine differentiation of prostate cancer cells [16, 17].

Here, we establish a microRNA expression profile of *in vitro* transdifferentiated LNCaP cells by androgen depletion in combination with global gene expression profiling. Furthermore, we show an impact of miR-17, -20a/b and -106a/b on the expression of their target gene CCND1 as well as on growth behavior and apoptosis of LNCaP cells to confirm the impact of miRNA deregulation during NETD of prostate cancer cells.

## Materials and methods

### Cell lines

The human embryonic kidney 293 cell line containing the SV40 T-antigen (HEK293T) and the human prostate cancer cell line LNCaP were purchased from the American Type Culture Collection (ATCC/LGC Standards GmbH, Wesel, Germany) May 2014. The number of passages between thawing and use in the described experiments was <10. HEK293T cells were cultivated in DMEM (Thermo Fisher Scientific, Schwerte, Germany) supplemented with 10% heat-inactivated FCS (Sigma Aldrich, Hamburg, Germany), Penicillin (100 U/ml) and Streptomycin (100 μg/ml). LNCaP cells were grown in RPMI 1640 without phenol red indicator supplemented with 10% heat-inactivated FCS, l-Glutamin (1 mM final concentration), Penicillin (100 U/ml) and Streptomycin (100 μg/ml). For *in vitro* neuroendocrine transdifferentiation of LNCaP cells, FCS was replaced by charcoal-stripped FCS (Sigma Aldrich, Hamburg, Germany) and LNCaP cells were cultivated for 14 days in androgen-free medium whereat medium was replaced every 72 h. For Mycoplasma testing cells cultured on coverslips in 12-well plates were fixed with methanol for 15 min, mounted on slides with VECTASHIELD^®^ mounting medium containing DAPI (Vector Laboratories, Burlingame,CA, USA) and examined with a fluorescence microscope (Nikon Eclipse Ts2, Tokia, Japan). Last Mycoplasma testing was carried out October 2017. Cell lines used were frequently examined regarding morphology, doubling time and growth.

### RNA extraction

RNA extraction from cell lines was performed using peqGOLD RNAPure (Peqlab, Erlangen, Germany). Total RNA was isolated according to the manufacturer's instructions, except washing of RNA with ethanol due to loss of miRNAs. For microarray analysis, total and short RNA isolation was carried out using miRNeasy Kit (Qiagen, Hilden, Germany).

### Microarray analysis

For gene expression profiling, hybridization experiments were performed and analyzed using 200 ng of total RNA for mRNA analysis on Affymetrix Gene Chips HG-U133 Plus 2.0 (54,000 probe sets for 47,400 transcripts and 38,500 genes, High Wycombe, UK) while miRNA labelling for the miRNA3.0 chip was done using 500 ng of total RNA and the Genesphere FlashTag labelling kit as described previously [18]. Total RNA expression of three different in vitro NETDs were analyzed using the Affymetrix Gene Chip Scanner 3000, the Affymetrix GeneChip Operating Software 1.4, and comparatively evaluated using the significance analysis of microarrays (SAM) approach [19]. Gene expression measures are available at GEO (https://www.ncbi.nlm.nih.gov/geo/query/acc.cgi?acc=GSE105416).

### Quantitative real-time PCR and reverse transcription PCR

For mRNA analysis, cDNA synthesis was performed with the High Capacity cDNA Reverse Transcription Kit (Applied Biosystems, Darmstadt, Germany) using 1 μg of total RNA and RT- random primers. RT-PCRs were performed with sequence-specific primers in a final volume of 50 μl containing 50 ng cDNA using standard PCR protocol as follows: 95°C for 5 min followed by 36 cycles of 95°C for 30 sec, 60°C for 45 sec and 72°C for 45 sec. Beta-actin and GAPDH served as endogenous controls. PCR products were subsequently separated on a 2% agarose gel containing GelRed® Nuleic Acid Gel Stain (Biotium Inc., Fremont CA, USA) and were visualized by ultraviolet light. qRT-PCRs were performed with the iQ5 real-time PCR detection system (BioRad, Munich, Germany) using sequence-specific primers and VeriQuest SYBR Green qPCR Master Mix (Affymetrix, Schwerte, Germany) according to the manufacturer’s protocols. All qRT-PCRs were measured in duplicates in a final volume of 25 μl containing 50 ng cDNA. The thermal cycling conditions were as follows: 95°C for 10 min followed by 45 cycles of 95°C for 15 sec, 58°C for 30 sec and 60°C for 45 sec. For quality control, melting curve analysis was performed. Calculation of relative mRNA expression was carried out using the ΔΔCt method with 18sRNA as endogenous reference.

For miRNA analysis, 1 μg total RNA was reverse transcribed using miScript PCR Starter Kit (Qiagen, Hilden, Germany) according to the manufacturer's instructions. qRT-PCRs were performed on the VeriQuest SYBR Green qPCR Master Mix (Affymetrix, Schwerte, Germany) using sequence-specific forward primers and 10x miScript Universal Primer (Qiagen, Hilden, Germany) as reverse primer with following thermal cycling conditions: 95°C for 10 min followed by 45 cycles of 95°C for 15 sec, 58°C for 30 sec and 60°C for 45 sec. For quality control, melting curve analysis was performed. To quantify the miRNA expression after NETD, we used the relative quantification (ΔΔCt) method with 5.8sRNA serving as an internal control. The RT-PCR primers and qRT-PCR primers for detection of mRNA and miRNA expression are listed in S1 Table.

### Target prediction

MiRNA target prediction was carried out using TargetScan (release 7.1; http://www.targetscan.org/)

### Plasmids

The miRNA expression plasmids were generated by PCR amplification of nucleotides 91,350,335–91,350,756 of chromosome 13 for miR-17, 91,350,951–91,351,273 of chromosome 13 for miR-20a, 134,169,622–134,170,000 of X chromosome for miR-20b, 134,169,722–134,170,440 of X chromosome for miR-106a and nucleotides 100,093,902–100,094,174 of chromosome 7 for miR-106b from human genomic DNA and by insertion into the pSG5 vector (Agilent technologies, Ratingen, Germany). Overexpression of the corresponding miRNAs after transfection in HEK293T and LNCaP cells was verified by qRT-PCR (S1 Fig). The nucleotides 2005–3089 of the CCND1 mRNA (accession number: NM_053056.2) containing a part of the corresponding 3’UTR were amplified via PCR using specific primers from human genomic DNA and inserted into pMIR-RNL-TK reporter vector which is described elsewhere [20]. The mutagenesis of the predicted target site seed sequences of pMIR-RNL-TK reporter constructs were performed with QuickChange Site Directed Mutagenesis Kit (Stratagene, Heidelberg, Germany), following the instructions of the manufacturer's manual. The primer sequences used for cloning and site directed mutagenesis are shown in S1 Table.

### Dual-luciferase assay

For Luciferase Reporter Assays, 2×10^5^ HEK293T were seeded per well in 24-well plates. After 24 h, cells were transfected with 0.8 μg of expression plasmid and 0.2 μg reporter plasmid using Polyfect Transfection Reagent (Qiagen, Hilden, Germany). Luciferase reporter assays were performed 48 h after transfection using the Dual-Luciferase Reporter Assay System in accordance with the manufacturer's instructions (Promega, Mannheim, Germany).

### Western blotting

For transfection of LNCaP cells, 6×10^5^ cells were cultivated in 6-well plates. After 24 h, cells were transfected with 2 μg of expression plasmid DNA using jetPRIME^®^ transfection reagent (Polyplus transfection, Sélestat, France). Cells were lysed 48 h after transfection with 2× sample buffer (130 mmol/l Tris/HCl, 6% SDS, 10% 3-mercapto-1,2-propandiol, 10% glycerol, and 0.05% bromophenol blue). 30 μg of extracted proteins were separated by 9% Tricine-SDS-Polyacrylamide-Gel electrophoresis and transferred to a nitrocellulose membrane (Whatman, GE Healthcare, Freiburg, Germany) by electroblotting. For immune detection the primary antibodies anti-CCND1 monoclonal rabbit antibody (clone SP4, Sigma-Aldrich, Hamburg, Germany) and anti-ß-actin monoclonal HRP antibody (clone AC15, Sigma-Aldrich, Hamburg, Germany) were used. Secondary goat anti-rabbit HRP clone 31460 antibody was purchased from Pierce (Thermo Fisher Scientific, Schwerte, Germany). Bands were visualized by ECL plus Western Blotting Substrate from Pierce (Thermo Fisher Scientific, Schwerte, Germany) and the Fujifilm LAS-3000 gel documentation system (Kleve, Germany) by densitometrical quantification.

### Colony formation assay

6x10^5^ LNCaP cells were seeded in 6-well plates and transfected with 2 μg expression plasmid DNA using jetPRIME (Polyplus transfection, Sélestat, France). 24 hours after transfection, cells were detached by trypsin, resuspended in medium, seeded in 6-well plates (2500 cells/well) and cultured for 12 additional days. After medium replacement cultures were stained with 0.4% cristal violet, fixed with 4% paraformaldehyde for 30 minutes and washed 3 times with PBS. Wells were photographed and densitometrically analyzed by ImageJ 1.48v (National Institute of Health, Bethesda, USA).

### Cell proliferation assay

3x10^5^ LNCaP cells were seeded in 6-well plates, transfected with 2 μg expression plasmid DNA and cultivated for 24–72 h. For measuring cell numbers on day 0 to 3 after transfection, cells were detached with trypsin and resuspended in 1 ml medium. Cell numbers were determined by CASY 1 cell counter (Schärfe System, Reutlingen, Germany).

### Flow cytometric determination of apoptosis by annexin V-FITC/propidium iodide double staining

Cells were collected three days after transfection with different miRNAs. Subsequently, 100,000 cells were stained with 5 μl Annexin V-FITC (Immunotools, Friesoythe, Germany) diluted in 195 μl DMEM for 20 min on ice. After washing with cold DMEM, 200 μl of the propidium iodide solution (5 μg/ml PI diluted with DMEM) was added to the samples and the acquisition was directly performed utilizing a FACScalibur System and analyzed by Cell Quest software (Becton Dickinson, Franklin Lakes, USA).

### Gene ontology and pathway enrichment analysis

For functional annotation of deregulated genes after NETD of LNCaP cells, we used DAVID, which consists of an integrated biological knowledgebase and analytic tools [21, 22]. We analyzed each 1000 most induced and repressed genes and identified the over-represented gene ontology (GO) categories in biological processes with the p-value <0.05 and count >2. In addition, DAVID was also applied for identifying the significant pathways using cut-off criteria of p-value <0.05 and count >2. p-values represent the EASE score from a modified, more conservative Fisher Exact test, which is integrated in DAVID.

### Data analysis and statistical methods

Statistical evaluation of the luciferase assays, real-time qRT-PCRs, colony formation, cell proliferation and apoptosis assays were performed with SigmaPlot 10 (Systat, Erkrath, Germany) using Student’s t-test analysis. All statistical tests were performed as two-sided and p-values of <0.05 were considered as significant. Western blots were quantified by ImageJ 1.48v (National Institute of Health, Bethesda, USA).

## Results

### In vitro NETD of LNCaP cells by androgen depletion

**Fig 1:**
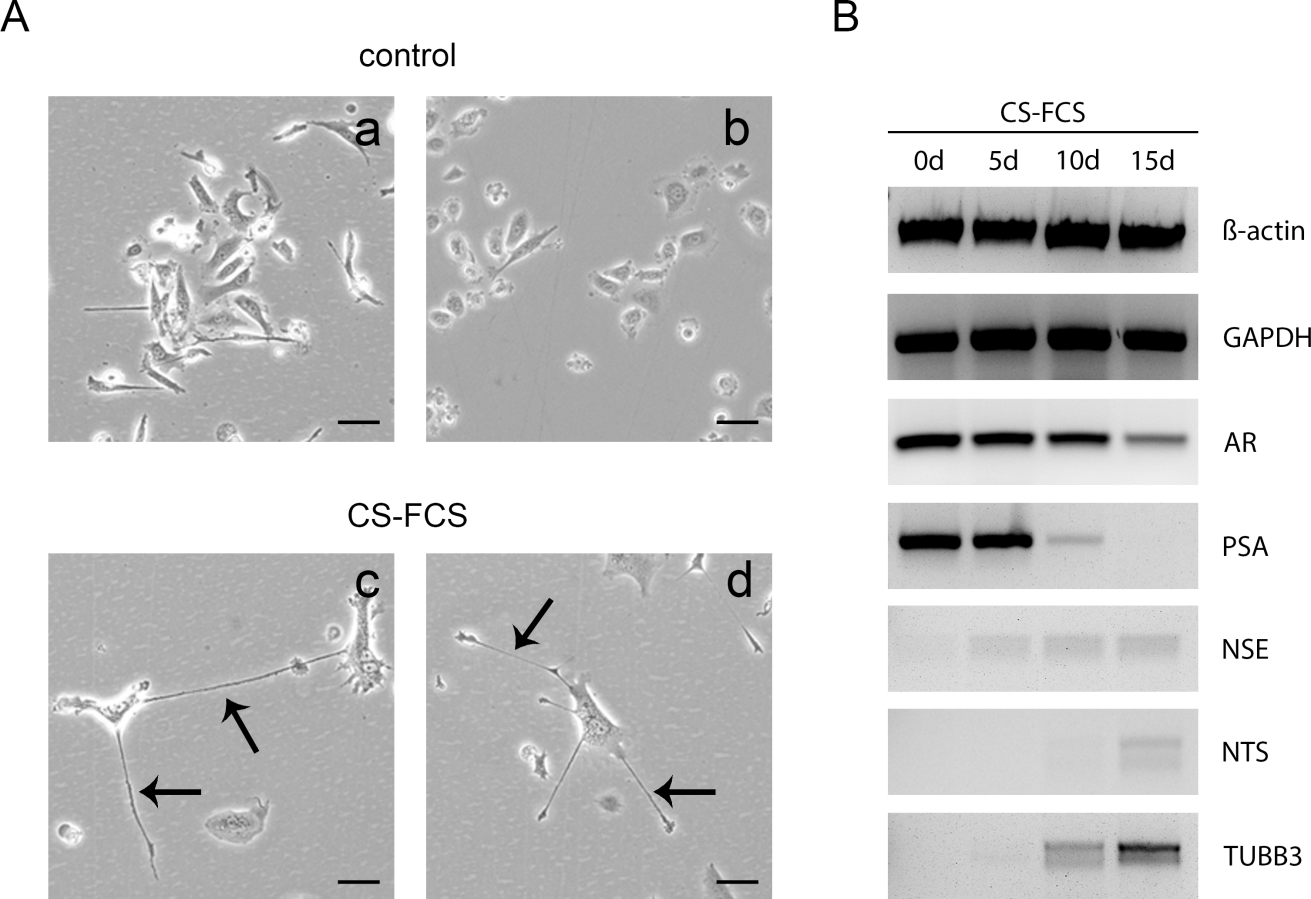
Neuroendocrine transdifferentiation of LNCaP cells through androgen deprivation. (A) LNCaP cells were cultivated in media with charcoal-stripped FCS (CS-FCS) or control FCS for 14 days. Transdifferentiated cells show dendrite like cell processes indicated by black arrows. (B) Successful transdifferentiation was validated through time course expression analysis of AR, PSA, NSE, NTS and TUBB3 in transdifferentiated LNCaP cells compared to untreated cells during androgen deprivation for 14 days by RT-PCR. Beta-actin and GAPDH served as endogenous controls.

To induce NETD in the prostatic LNCaP cell line, we cultivated LNCaP cells for 14 days in medium with charcoal-stripped FCS. The induction of NETD in LNCaP cells by androgen depletion is well described and represents a commonly used model for prostatic NETD [23]. After 14 days, cells showed morphological changes with neurite-like outgrowths accompanied by a decrease of cell growth (Fig 1A). To confirm NETD we examined marker gene expression in a time course experiment including androgen receptor (AR), neuron-specific enolase (NSE), prostate-specific antigen (PSA), neurotensin (NTS) and tubulin beta III (TUBB3). RT-PCR results of amplificated transcript fragments show after switch to androgen-deficient medium the previously described decrease of AR and PSA expression as well as an induction of NSE, NTS and TUBB3 during cultivation for 2 weeks (Fig 1B). These findings confirm a successful NETD allowing subsequent gene array analysis of NE-like LNCaP cells.

### NETD of LNCaP cells results in global gene and miRNA expression changes

**Fig 2:**
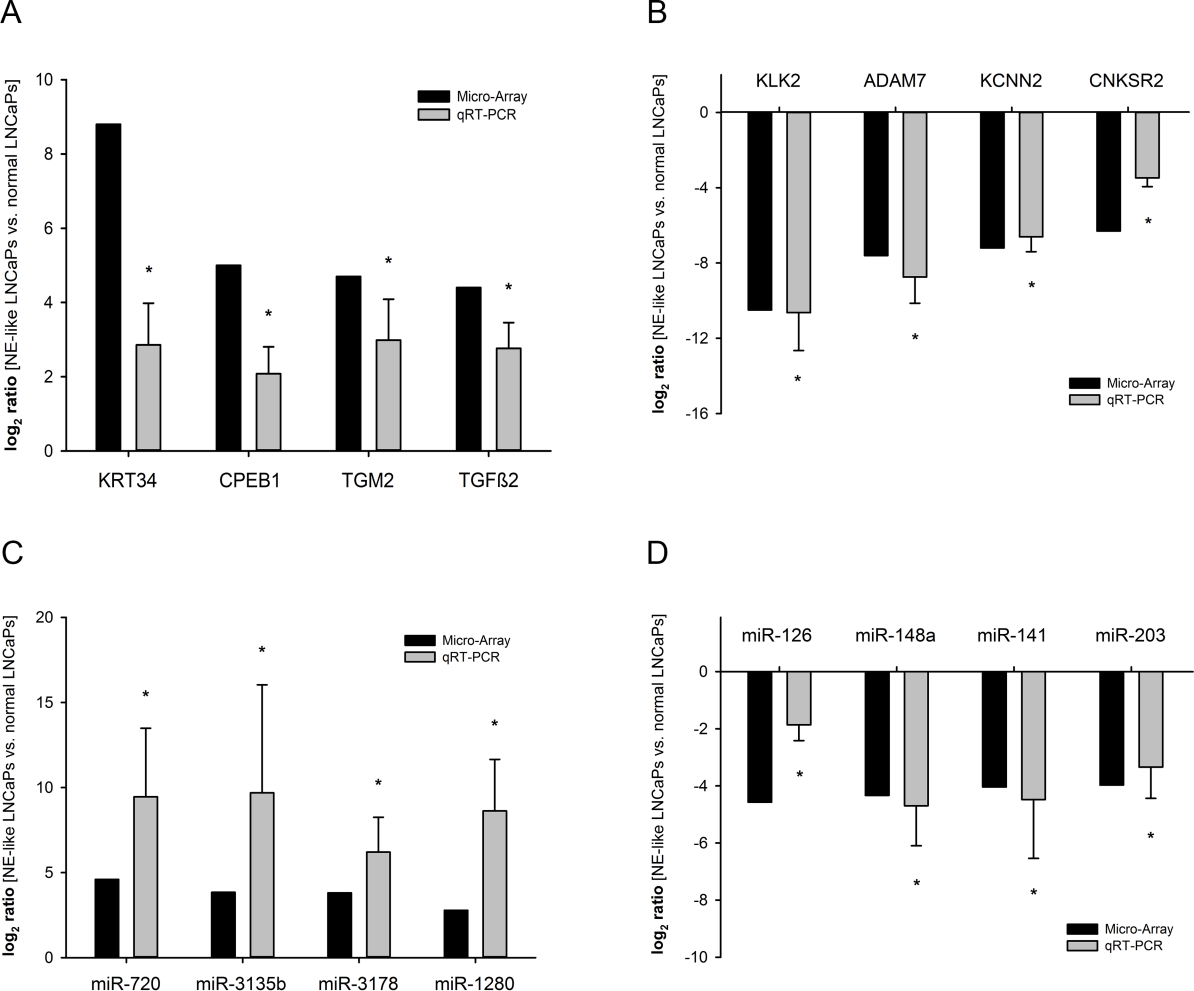
Validation of differentially expressed genes in NE-differentiated LNCaP cells compared to untreated cells. The expression of eight mRNAs (A, B) and miRNAs (C, D) that were assumed to be elevated or reduced according to their signals in microarray (black bars) was assessed by qRT-PCR (grey bars). Results represent the mean from 4 independent NE-transdifferentiations performed in duplicates (*, p<0.05).

We performed gene array analysis of NE-like LNCaP cells pooled from 3 independent NETDs to determine expression changes of mRNA and miRNA in treated LNCaP cells. NETD of LNCaP cells resulted in a wide significant change of mRNA and miRNA expression. Table 1 shows the 20 most induced and repressed mRNAs after NETD. The strongest upregulated genes were KRT34, SCG5 and GPR115. Conversely, KLK3 (PSA), KLK2 and ADAM7 were downregulated. Furthermore, we found 87 miRNAs with at least 2-fold upregulation and 85 miRNAs with at least 2-fold downregulation. The most differentially expressed miRNAs were miR-720, miR-3135b, miR-3178 and miR-424* for induced miRNAs and miR-126, miR-148a, miR-203 as well as miR-203 for the downregulated ones (Table 2). To validate the reliability of the microarray data qRT-PCR was used to assess the expression levels of each eight deregulated mRNA and miRNA in additional four independent NETD LNCaP cultures not used for microarray analysis. We examined the expression of KRT34, CPEB1, TGM2 and TGFB2 for induced genes (Fig 2A) as well as KLK2, ADAM7, KCNN2 and CNKSR2 for repressed mRNAs, which all are regulated independent of androgen receptor signaling (Fig 2B). For miRNA array validation we chose the mostly deregulated miR-720, miR-3135b, miR-3178 and miR-1280 for induced miRNAs (Fig 2C) and miR-126, miR-148a, miR-141 and miR-203 for repressed miRNAs (Fig 2D). The qRT-PCR results show a high degree of concordance to the expression changes observed in gene arrays, verifying the reliability of the array data.

**Table 1:** Differentially expressed genes in neuroendocrine differentiated LNCaP cells compared to untreated LNCaP cells.

Gene title	Gene symbol	Ratio (log_2_)
kallikrein-related peptidase 3	KLK3	-10.8
kallikrein-related peptidase 2	KLK2	-10.5
ADAM metallopeptidase domain 7	ADAM7	-7.6
NK3 homeobox 1	NKX3-1	-7.5
potassium intermediate/small conductance calcium-activated channel, subfamily N, member 2	KCNN2	-7.2
transmembrane protein with EGF-like and two follistatin-like domains 2	TMEFF2	-6.9
mucin 5B, oligomeric mucus/gel-forming	MUC5B	-6.8
family with sequence similarity 20, member A	FAM20A	-6.5
male germ cell-associated kinase	MAK	-6.4
connector enhancer of kinase suppressor of Ras 2	CNKSR2	-6.3
microseminoprotein, beta-	MSMB (PSP94)	-6.2
regulatory factor X, 6	RFX6	-6.2
KIAA1210	KIAA1210	-6.1
thymosin beta 15a / thymosin beta 15B	TMSB15A/TMSB15B	-6.0
glutamate receptor, ionotropic, N-methyl-D-aspartate 3A	GRIN3A	-5.6
fibroblast growth factor 9 (glia-activating factor)	FGF9	-5.5
neuropilin (NRP) and tolloid (TLL)-like 1	NETO1	-5.5
keratin associated protein 9–9	KRTAP9-9	-5.4
transmembrane protease, serine 2	TMPRSS2	-5.4
AF4/FMR2 family, member 3	AFF3	-5.4
keratin 34	KRT34	8.8
secretogranin V (7B2 protein)	SCG5	7.2
G protein-coupled receptor 115	GPR115	6.4
keratin 33A	KRT33A	5.9
keratin associated protein 1–5	KRTAP1-5	5.3
keratin 7	KRT7	5.2
cytoplasmic polyadenylation element binding protein 1	CPEB1	5.0
interleukin 13 receptor, alpha 2	IL13RA2	4.8
neutrophil cytosolic factor 2	NCF2	4.8
transglutaminase 2 (C polypeptide, protein-glutamine-gamma-glutamyltransferase)	TGM2	4.7
aldo-keto reductase family 1, member B10 (aldose reductase)	AKR1B10	4.6
insulin-like growth factor binding protein 3	IGFBP3	4.6
Rho GTPase activating protein 27	ARHGAP27	4.6
sidekick cell adhesion molecule 2	SDK2	4.6
RUN and FYVE domain containing 2	RUFY2	4.6
nervous system abundant protein 11	NSAP11	4.5
CD33 molecule	CD33	4.4
transforming growth factor, beta 2	TGFB2	4.4
adhesion molecule with Ig-like domain 2	AMIGO2	4.4
keratin 80	KRT80	4.4

**Table 2:** Differentially expressed miRNAs in neuroendocrine differentiated LNCaP cells compared to untreated LNCaP cells.

miRNA	Ratio (log_2_)	miRNA	Ratio (log_2_)
hsa-miR-720	4.62	hsa-miR-126	-4.57
hsa-miR-3135b	3.84	hsa-miR-148a	-4.33
hsa-miR-3178	3.81	hsa-miR-141	-4.03
hsa-miR-424*	3.56	hsa-miR-203	-3.97
hsa-miR-23a*	3.48	hsa-miR-18b	-3.96
hsa-miR-29b-1*	3.09	hsa-miR-18a	-3.88
hsa-miR-27a*	2.88	hsa-miR-3613-5p	-3.41
hsa-miR-1280	2.78	hsa-miR-497	-3.40
hsa-miR-1296	2.62	hsa-miR-4521	-3.19
hsa-miR-378g	2.58	hsa-miR-301a	-2.99
hsa-miR-139-5p	2.45	hsa-miR-34a	-2.83
hsa-miR-4656	2.43	hsa-miR-1246	-2.75
hsa-miR-3615	2.39	hsa-miR-17*	-2.60
hsa-miR-1260	2.28	hsa-miR-4668-5p	-2.47
hsa-miR-663	2.26	hsa-miR-29c	-2.40
hsa-miR-664*	2.20	hsa-miR-374b	-2.38
hsa-miR-504	2.19	hsa-miR-148b	-2.37
hsa-miR-4665-5p	2.13	hsa-miR-3613-3p	-2.32
hsa-miR-378d	2.12	hsa-miR-20b	-2.25
hsa-miR-3195	2.12	hsa-miR-105	-2.25
hsa-miR-3609	2.09	hsa-miR-1287	-2.19
hsa-miR-4467	2.01	hsa-miR-25*	-2.04
hsa-miR-4667-5p	1.96	hsa-miR-20a	-1.96
hsa-miR-378f	1.95	hsa-miR-26b	-1.95
hsa-miR-4454	1.94	hsa-miR-93*	-1.92
hsa-miR-193a-5p	1.83	hsa-miR-3935	-1.88
hsa-miR-181b	1.82	hsa-miR-195	-1.86
hsa-miR-4443	1.80	hsa-miR-3065-5p	-1.84
hsa-miR-3197	1.76	hsa-miR-200a*	-1.83
hsa-miR-30b*	1.76	hsa-miR-15a	-1.82
hsa-miR-122	1.74	hsa-miR-330-3p	-1.80
hsa-miR-422a	1.71	hsa-miR-106a	-1.79
hsa-miR-221*	1.64	hsa-miR-106b	-1.78
hsa-miR-1260b	1.62	hsa-miR-17	-1.77
hsa-mir-3676	1.61	hsa-miR-1202	-1.65
hsa-miR-494	1.59	hsa-mir-548a	-1.60
hsa-miR-197	1.55	hsa-miR-660	-1.58
hsa-miR-4508	1.55	hsa-miR-200c	-1.55
hsa-miR-181a-2*	1.52	hsa-miR-339-5p	-1.53
hsa-miR-378i	1.50	hsa-miR-1180	-1.53
hsa-miR-181a	1.49	hsa-miR-500a	-1.49
hsa-miR-320e	1.44	hsa-miR-128	-1.47
hsa-miR-4492	1.43	hsa-miR-21*	-1.45
hsa-miR-378	1.40	hsa-miR-4324	-1.45
hsa-miR-378*	1.40	hsa-miR-1269	-1.44
hsa-miR-505*	1.39	hsa-mir-200c	-1.41
hsa-miR-378e	1.36	hsa-miR-19a	-1.38
hsa-miR-181c	1.36	hsa-mir-425	-1.36
hsa-miR-196a	1.36	hsa-miR-2392	-1.35
hsa-miR-1908	1.34	hsa-miR-93	-1.30

### GO enrichment analysis of the interaction network

GO analysis is a commonly used approach for functional analysis of large-scale genomic or transcriptomic data [24]. To investigate the function changes leading to NETD of LNCaP cells, we used the online tool DAVID to identify over-represented GO categories in biological process with EASE score p-value less than 0.05 and count larger than 2 as threshold. Several GO categories were enriched among these genes in the regulatory network. In Table 3 we list the top 20 GO biological process categories for repressed (Table 3A) and induced genes (Table 3B). Most enriched GO terms of repressed genes were related to “oxidation-reduction process”, “cell division”, “mitotic nuclear division”, and “cell proliferation”, as shown in Table 3A leading to the post-mitotic phenotype of NE-like prostate cancer cells. The GO terms for induced genes were involvement in “regulation of transcription from RNA polymerase II promoter” and “signal transduction”.

**Table 3:** Classification of deregulated genes in LNCaP after NETD according to GO terms with p-value <0.05. (A) GO enrichment results for repressed genes (Top 20). (B) GO enrichment results for induced genes (Top 20).

	GO description	Count	p-value
**A**	oxidation-reduction process	35	1,20E-03
	cell division	31	1,70E-06
	mitotic nuclear division	27	1,90E-07
	cell proliferation	25	1,10E-03
	proteolysis	25	4,10E-02
	cell adhesion	23	4,90E-02
	cellular protein metabolic process	19	5,50E-08
	CENP-A containing nucleosome assembly	18	8,90E-15
	sister chromatid cohesion	18	3,90E-08
	positive regulation of gene expression	18	6,00E-03
	double-strand break repair via nonhomologous end joining	17	1,30E-10
	negative regulation of gene expression, epigenetic	16	3,60E-11
	nucleosome assembly	16	8,10E-06
	positive regulation of gene expression, epigenetic	15	9,70E-09
	telomere capping	14	3,80E-14
	telomere organization	14	5,90E-13
	DNA-templated transcription, initiation	14	5,10E-11
	gene silencing by RNA	14	7,00E-05
	chromosome segregation	13	1,90E-06
	regulation of cell cycle	12	2,60E-03
**B**	positive regulation of transcription from RNA polymerase II promoter	60	5,20E-05
	signal transduction	56	1,80E-02
	negative regulation of transcription from RNA polymerase II promoter	38	1,60E-02
	positive regulation of cell proliferation	35	6,20E-05
	positive regulation of transcription, DNA-templated	35	4,10E-04
	oxidation-reduction process	35	4,20E-03
	negative regulation of apoptotic process	33	2,00E-04
	cell adhesion	32	5,00E-04
	negative regulation of transcription, DNA-templated	28	2,00E-02
	negative regulation of cell proliferation	27	2,00E-03
	inflammatory response	23	1,70E-02
	angiogenesis	21	1,40E-04
	cell-cell signaling	21	7,70E-04
	positive regulation of gene expression	20	2,70E-03
	cellular protein metabolic process	17	4,30E-06
	extracellular matrix organization	17	1,70E-03
	positive regulation of angiogenesis	16	1,40E-05
	nucleosome assembly	16	2,10E-05
	positive regulation of cell migration	16	2,40E-03
	small GTPase mediated signal transduction	16	3,00E-02

### Pathway enrichment analysis of the interaction network

**Fig 3:**
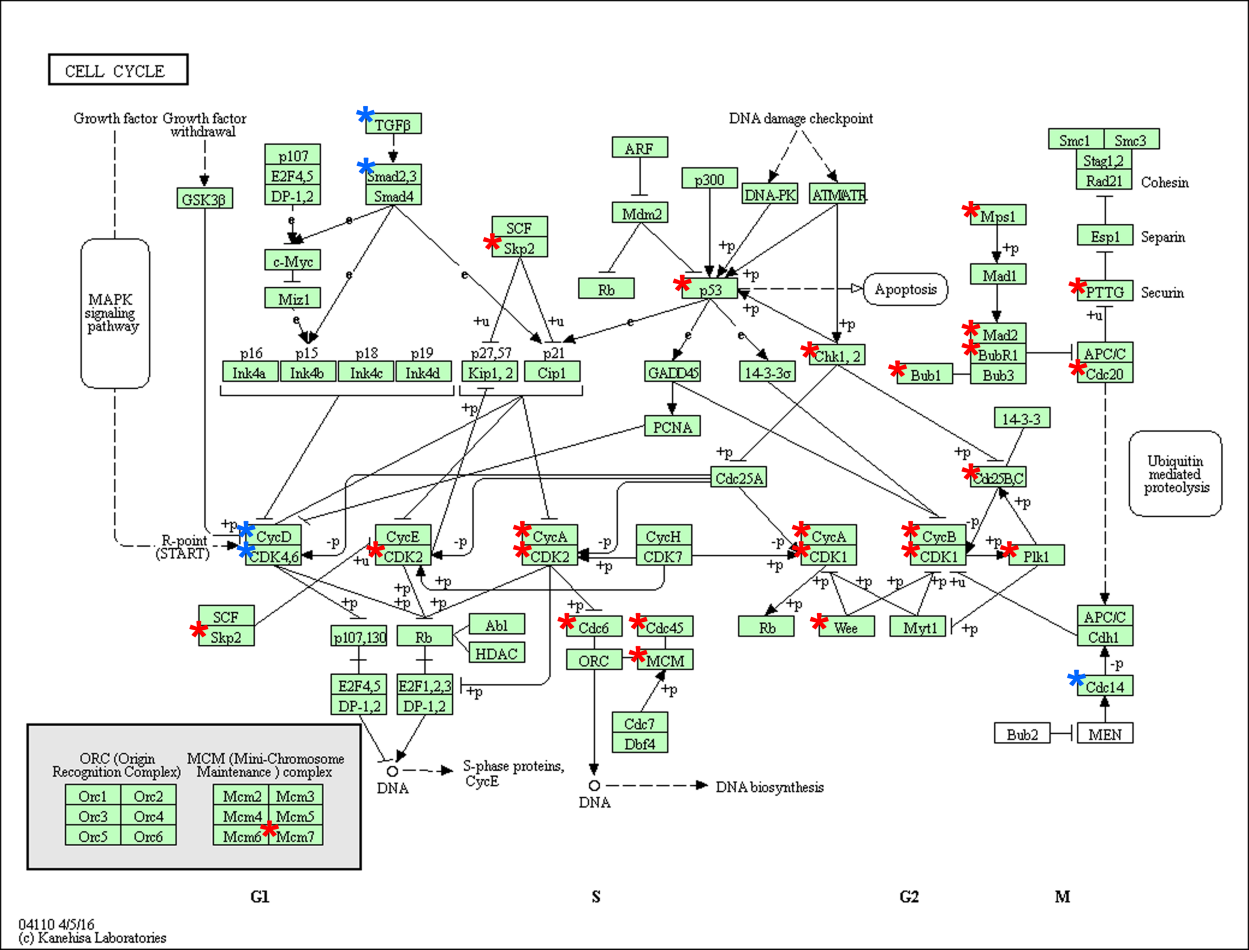
Deregulated genes in the KEGG pathway “cell cycle” in LNCaP cells after NETD. The pathway enrichment result for “cell cycle” (hsa04110) shows repressed genes marked with red asterisks and induced genes marked with blue asterisks.

To gain further insights into the function of genes in the interaction network, we used DAVID to analyze the pathway enrichment. The results indicate a multitude of involved pathways for repressed and induced genes listed in Table 4A and 4B. Interestingly we found an enrichment of repressed genes in PI3K-Akt signaling, p53 signaling as well as cell cycle and prostate cancer (Table 4A). Otherwise, most enriched pathways for induced genes were pathways in cancer, TNF signaling and TGFB signaling (Table 4B). Fig 3 shows cell cycle pathway with repressed (red asterisks) and induced (blue asterisks) genes (Fig 3). Several involved cyclins (CycA, CycB), cyclin dependent kinases (CDK1, CDK2) and cell division cycle genes (Cdc6, Cdc20, Cdc45) are downregulated after NETD whereas one cyclin CCND1 (CycD), CDK4,6 and TGFB are induced.

**Table 4:** Pathway analysis of deregulated genes in LNCaP after NETD according with p-value <0.05. (A) Pathway enrichment results for repressed genes. (B) Pathway enrichment results for induced genes.

	Pathway	Count	p-value
**A**	Metabolic pathways	55	4,80E-02
	Viral carcinogenesis	24	7,90E-07
	PI3K-Akt signaling pathway	20	3,70E-02
	Systemic lupus erythematosus	17	1,80E-05
	Alcoholism	17	5,10E-04
	Biosynthesis of antibiotics	14	3,80E-02
	Transcriptional misregulation in cancer	12	3,60E-02
	p53 signaling pathway	10	5,40E-04
	Progesterone-mediated oocyte maturation	10	3,50E-03
	Cell cycle	10	3,20E-02
	FoxO signaling pathway	10	4,80E-02
	Protein digestion and absorption	9	1,20E-02
	Arginine and proline metabolism	8	1,80E-03
	ECM-receptor interaction	8	3,40E-02
	Prostate cancer	8	3,60E-02
	Arrhythmogenic right ventricular cardiomyopathy (ARVC)	7	3,90E-02
	Pentose and glucuronate interconversions	6	8,30E-03
	Glycine, serine and threonine metabolism	5	4,80E-02
**B**	Pathways in cancer	25	2,10E-02
	Viral carcinogenesis	18	3,00E-03
	Alcoholism	17	1,60E-03
	Systemic lupus erythematosus	14	2,30E-03
	Hematopoietic cell lineage	10	6,10E-03
	TNF signaling pathway	10	2,40E-02
	TGF-beta signaling pathway	9	1,70E-02
	Malaria	8	2,90E-03
	Cytosolic DNA-sensing pathway	8	1,30E-02
	Legionellosis	7	1,90E-02
	NOD-like receptor signaling pathway	7	2,10E-02

### Inhibition of cyclin D1 expression by the miR-17 microRNA family

**Fig 4:**
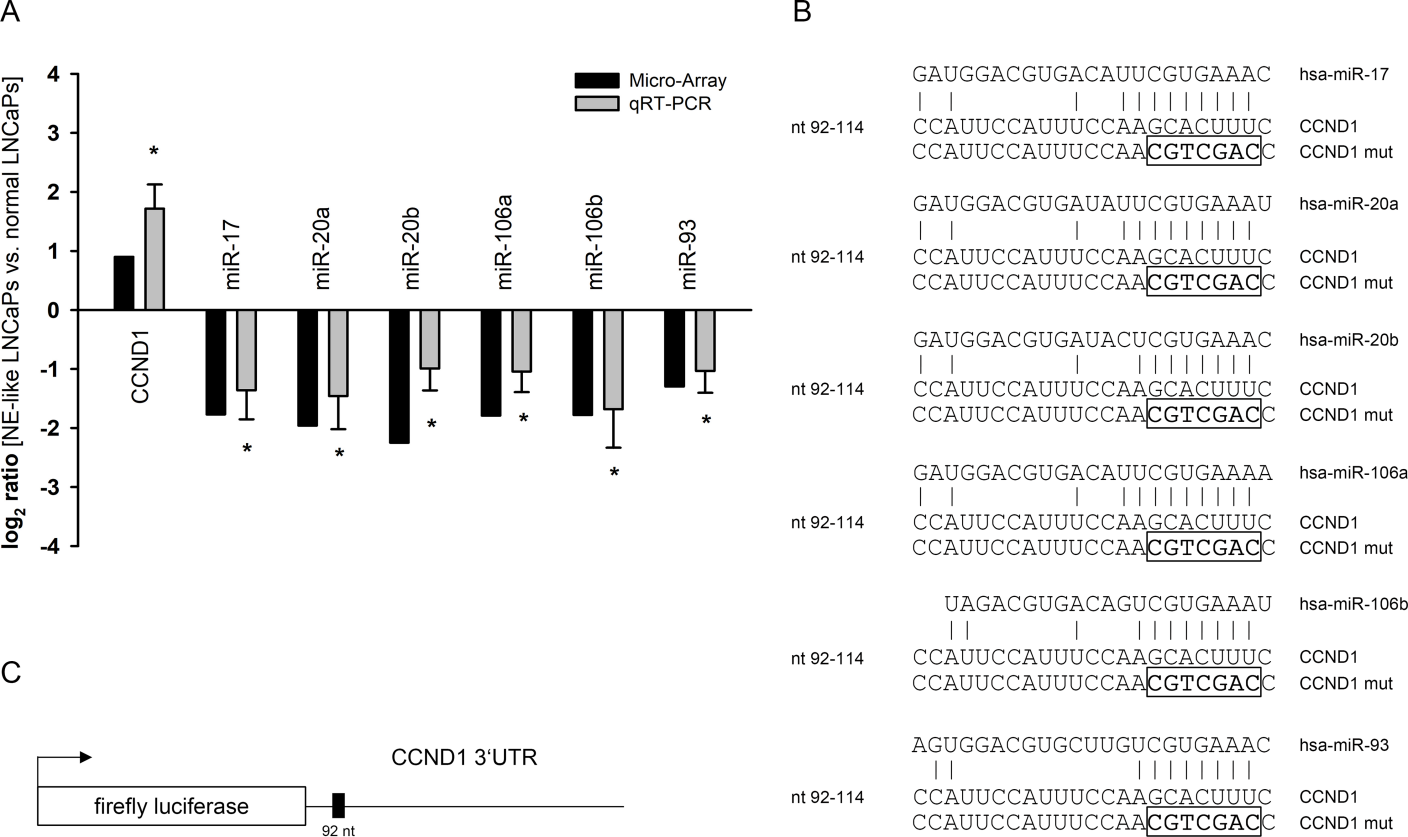
Quantification of CCND1 and miRNA expression in neuroendocrine transdifferentiated LNCaP cells compared to untreated LNCaP cells and predicted miRNA target sites. The expression of CCND1 and miR-17 family miRNAs (A) that were assumed to be elevated or reduced according to their signals in microarray (black bars) was assessed by qRT-PCR (grey bars). CCND1 was predicted to be elevated while miR-17, miR-20a, miR-20b, miR-106a, miR-106b and miR-93 were predicted to be reduced in NE-transdifferentiated LNCaP as compared to untreated cells (*, p<0.05). (B) A schematic representation of the predicted miRNA interaction site and the mutated seed sequences are shown. (C) The 3’UTR region of CCND1 is depicted.

**Fig 5:**
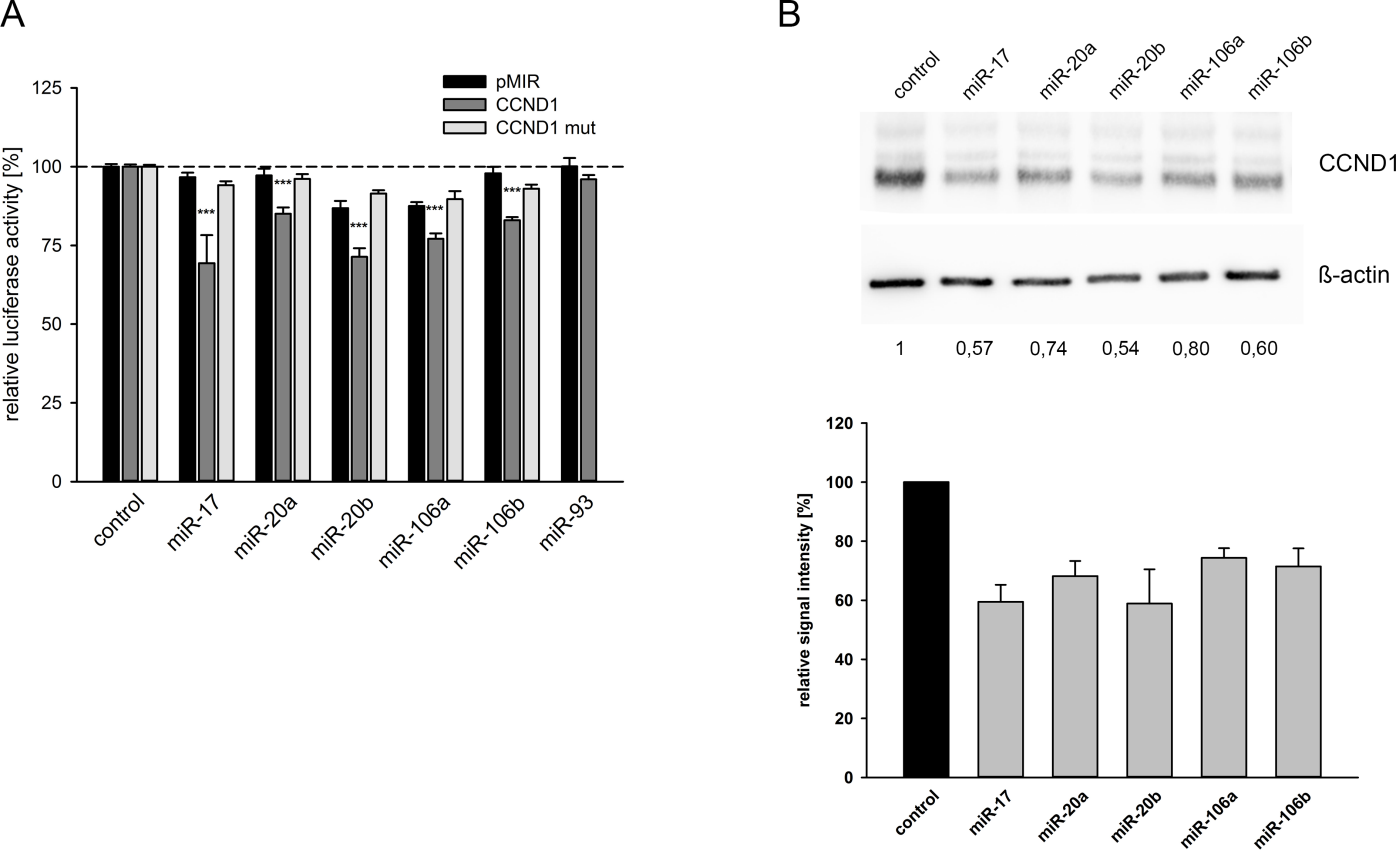
Response of CCND1 3’UTR and protein expression towards miR-17 family. (A) The CCND1 3’UTR was cloned behind the luciferase reporter gene of the pMIR vector and the potential binding site for the indicated miRNAs in the 3’UTR was additionally mutated by site directed mutagenesis (CCND1 mut). The reporter gene construct was expressed with the miRNA expression construct or with the empty pSG5 vector as control in the indicated combinations. Results represent the mean of at least four independent experiments performed in duplicates. The dashed line represents the luciferase activity of the empty luciferase reporter plasmid with the empty pSG5 vector which was set to 100% (***, p<0.001). (B) LNCaP cells were transfected either with control vector or miRNA expression vectors. 48 hours post-transfection the protein expression of CCND1 was determined by Western blot using ß-actin as loading control. The densitometrical quantification of Western Blots represents the relative downregulation of CCND1 expression as determined in three independent experiments in relation to the corresponding ß-actin band as loading control.

The analysis of microarrays results for miRNA expression showed a deregulation of the miR-17 miRNA family. We observed a downregulation of miR-17, miR-20a, miR-20b, miR-106a, miR-106b as well as miR-93 (Table 2). A subsequent target prediction analysis using TargetScan identified cyclin D1 (CCND1) as a potential target gene included in the deregulated cell cycle pathway after NETD of LNCaP cells (Fig 3). To initially verify the decreased expression of these miRNAs and the corresponding induction of CCND1 after NETD, we performed qRT-PCR in additional four independent NETD LNCaP cultures confirming significant converse expression changes of the miRNA family and CCND1 in transdifferentiated LNCaP cells compared to normal LNCaP cells further supporting the hypothesis of CCND1 being a regulative target of the miR-17 family (Fig 4A). The predicted miRNA interaction sites inside the 3’UTR region of CCND1 are schematically shown in Fig 4B. The CCND1 3’UTR fragment containing the putative miRNA target site was inserted into a luciferase reporter vector (Fig 4C) and was co-transfected with miRNA expression vectors into HEK293T cells. All co-transfected miRNAs except miR-93 significantly reduced the activity of the luciferase reporter gene (p<0.05) under the regulatory control of the CCND1 3’UTR fragment compared to the empty reporter gene vector (Fig 5A). We next removed the seed sequence of the potential miRNA binding site, which is crucial for the miRNA-target mRNA interaction, by site-directed mutagenesis. The nucleotide exchange inside the miRNA target sequence is depicted in Fig 4B. Mutation in the binding site of the 3’UTR resulted in loss of responsiveness towards the targeting miRNAs (Fig 5A). To investigate the regulative capabilities of miR-17 miRNA family members, which showed an effect in luciferase assays, regarding the endogenous CCND1 protein we overexpressed the corresponding miRNAs in LNCaP cells and analyzed CCND1 expression level by Western Blot. Ectopic expression of each miRNA resulted in a reduction of CCND1 protein level (Fig 5B). We observed the strongest response for miR-17 and miR-20b inhibiting the expression of CCND1 to 57% and 54%, respectively.

As a second putative target mRNA for deregulated miRNAs after NETD, we additionally examined the interaction between highly repressed miR-148a (Table 1) and the 3’UTR of the corresponding predicted target TGFB2, an induced gene depicted in Fig 3. Luciferase assays as well as Western Blot analysis demonstrate the regulation of TGFB2 expression by miR-148a (S2 Fig), which was meanwhile published by Zhang and Li [25].

However, we demonstrate that CCND1 is directly and independently regulated by miR-17, miR-20a, miR-20b, miR-106a and miR-106b as members of the miR-17 miRNA family.

### Overexpression of miR-17 miRNA family reduces growth and induces apoptosis in LNCaP cells

**Fig 6:**
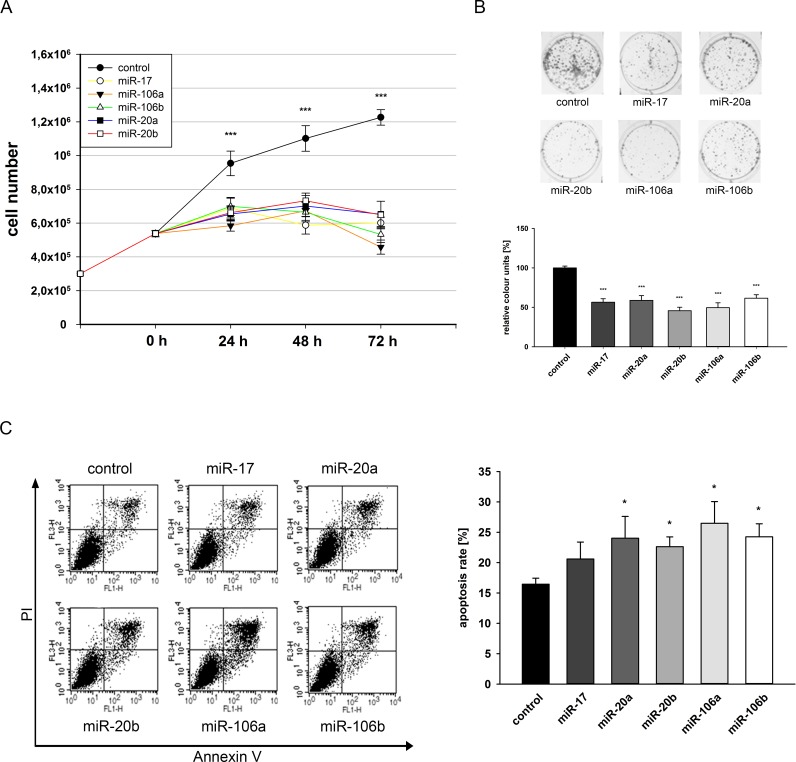
Effect of miR-17 family on cell growth, colony formation and apoptosis of LNCaP cells. LNCaP cells were transfected either with control vector or miRNA expression vectors and seeded in a limited cell number. Cell growth was determined by automated cell counting of parallel experiments at 24, 48 and 72 h post transfection for three independent experiments (A). Eight days after seeding, colonies were stained with crystal violet (B). Colony formation was quantified by densitometry analyses. Data show the mean and ± SEM of the densitometry analysis of three independent CFA experiments (***, p<0.001). For apoptosis analysis, LNCaP cells were harvested 72h post transfection and stained with Annexin-FITC/PI. In FACS analysis apoptosis was determined as sum of early and late apoptosis (upper/lower right quadrants). Dot blots display representative results (FL1-H: Annexin-FITC, FL3-H: PI), the right panel shows the mean of four independent experiments (C).

Finally, we analyzed the effect of previously used miR-17 miRNA family members on the growth behavior of LNCaP cells. Cells were seeded in 6-well plates and transfected with miRNA expressing vectors and the growth properties were assessed by counting cell numbers for 72 hours. Ectopic expression of each miRNA resulted in a reduced growth rate which was already significant after 24 hours post transfection (p<0.05) (Fig 6A). Next, we carried out colony formation assays with LNCaP cells after transfection of corresponding miRNAs. All five miRNAs significantly decreased the colony formation ability of LNCaP cells after 14 days by at least 40% (p<0.05) as shown in Fig 6B. To determine, whether the observed inhibition of cell growth and colony formation capacity after miRNA overexpression is a result of a putative apoptosis induction, we examined apoptosis of transfected LNCaP cells via Annexin V and PI staining and subsequent FACS analysis. Overexpression of miR-20a, miR-20b, miR-106a and miR-106b resulted in a significant induction of apoptosis (p<0.05) between 6% and 10% whereas miR-17 showed only a tendency of apoptosis induction (Fig 6C) compared to control LNCaP cells. Further experiments concerning modulation of LNCaP cell cycle after miRNA overexpression by PI staining and FACS analysis showed no significant alterations in the cell cycle distribution (Data not shown).

Our results demonstrate an impact of deregulated miR-17 miRNA family after NETD on LNCaP cell behavior based on growth induction and apoptosis inhibition.

## Discussion

Here we identified new genes and miRNAs that are deregulated after neuroendocrine transdifferentiation of LNCaP cells, which potentially play a role within this process. We demonstrate that neuroendocrine transdifferentiation of LNCaP cells by androgen deprivation leads to a wide change in gene and miRNA expression. NETD was accomplished by cultivation of LNCaP cells in androgen-free medium for 2 weeks leading to morphological and genetic changes as described in several studies [26]. We could detect the expected loss of PSA expression and a decrease of AR expression. Cerasuolo and colleagues demonstrated that NETD alters AR activation rather than AR gene expression [27]. Beside the observed decrease of AR expression in our case, a further loss of AR activity would explain the total loss of the AR dependent PSA expression. The increase of NSE expression is moderate but similar to the results of Martin-Orozco and colleagues [28]. The induced expression of TUBB3 and NTS during NETD was already reported by Zelivianski and Deeble and further confirms the successful transdifferentiation of the corresponding LNCaP cells [29, 30].

Beside the marker genes for NETD we found numerous genes which are repressed after NETD and whose expression is known to be AR dependent. For example NKX3-1 [31], TMPRSS2 [32] and MAK [33] are induced by androgens thus showing the expected repression after androgen depletion. Additionally, we confirmed the repression of p53 (0.2 fold) [34] and Ki67 (0.27 fold) [4]. Interestingly, we detected an upregulation of several keratins and keratin associated proteins (Table 1), whose function for tumorigenesis and progression is still not fully understood yet. Keratin 7 seems to be involved in metastasis in colon carcinoma [35].

Eder and colleagues observed gene expression changes in LNCaP cells after AR elimination by both blocking AR and androgen deprivation for 48 hours [36]. Terry and colleges showed that NETD and marker gene expression like PSA repression and NSE induction after androgen depletion is mostly observed after 7–14 days whereas further cultivation in androgen depleted medium leads to restoration of initial expression levels [37]. We can approve these findings showing explicit marker gene expression changes in LNCaP cells after 5–10 days cultivation in androgen-free growth medium and we further confirm the results of Eder and colleagues showing reduction of PCNA (0.6 fold), E2C (0.33 fold), DBI (0.5 fold) and IGFBP2 (0.03 fold) as well as induction of PIP5K1A (1.4 fold).

The mostly induced miRNAs after NETD were miR-720, miR-3135b, miR-3178 and miR-424* which are not described in connection with prostate cancer yet. Only miR-720 is known to be upregulated in recurrent prostate cancer after radical prostatectomy [38]. There are several publications concerning these miRNAs in other tumor entities as they are deregulated in breast cancer [39], colorectal cancer [40], cervical cancer [41] and have an impact on tumorigenesis, invasion and migration. On the contrary, we identified miR-126, miR-148a, miR-141 and miR-203 as mostly repressed miRNAs after NETD of LNCaP cells. Sun and colleges showed a decreased expression of miR-126 in prostate cancer, a negative correlation with aggressiveness as well as the pathological stage and thus a role in the malignant progression of prostate cancer [42]. An additional repression of miR-126 in NE-like cells would probably enhance the effects observed in prostate cancer tissue. MiR-148a is an androgen-responsive miRNA that promotes LNCaP prostate cell growth [43]. Additionally, we showed in former studies an upregulation of miR-148a in primary prostate cancer tissue compared to normal prostate tissue [11, 44], whereat the decrease in NE-like LNCaP likely results from lack of androgen and could be an explanation for the diminished cell proliferation of LNCaP after NETD. Similar to miR-148a, miR-141 is overexpressed in prostate cancer and castration resistant prostate cancer as well as enriched in blood sera of patients with advanced prostate cancer and it enhances growth of LNCaP cells [45]. MiR-203 is described as downregulated in prostate cancer and controls proliferation, migration and invasive potential of prostate cancer cell lines [46]. Thus, it is claimed as anti-metastatic whereat its downregulation would further increase metastatic properties [47]. Taken together, the top four repressed miRNAs seem to have an influence on cell proliferation of NE-like LNCaP cells as a characteristic of normal prostatic NE cells and NE-like tumor cells and on the other hand induce metastasis and invasion.

To gain insight into functions affected by NETD of LNCaP cells, GO and KEGG pathway analyses were performed to clarify possible biological functions and mechanisms altered after NETD, resulting in the identification of 189 GO terms and 26 deregulated pathways for each 1000 most induced and repressed genes. GO terms involving cell division and proliferation were significantly depleted which agreed with terms derived from the KEGG analysis. Looking at the cell cycle pathway, a multitude of genes, e.g. cyclins and cyclin dependent kinases, showed a diminished expression except Cyclin D1, CDK6 and members of the TGFB signaling pathway. The observed downregulation of cell cycle members probably leads to the described and observed reduced cell proliferation of differentiated LNCaP cells. In contrast, the upregulation of CCND1 presumably has no effect on cell cycle progression during NETD. Schiewer and colleges showed that CCND1 can act as a transcriptional repressor of AR target genes and inhibits DNA synthesis in LNCaP cells [48].

We demonstrate CCND1 as a target for the miR-17 family except miR-93 (miR-17, -20a/b, -106a/b), which are deregulated after NETD in LNCaP cells. MiR-17, -106a and -106b were found to be repressed down to 30%, miR-20a and -20b to 20–25% and miR-93 to 41% by microarray analysis. This deregulation could be confirmed for all six miRNAs by qRT-PCR for independent NETDs of LNCaP cells. The cause for the diminished expression of miR-17 family needs to be discussed. Guo and colleges showed that knockdown of AR decreased miR-17-92a cluster expression, containing miR-17, -18a, -19a/b, -20a and miR-92 family, in LNCaP cells as well as other AR-positive prostate cancer cell lines [49]. Moreover, it is known that miR-17-92a cluster is regulated by proto-oncogene MYC and thus a decreased MYC expression, as detected in NE-like LNCaP cells by microarray (repressed 0.6 fold after NETD), would result in the observed miR-17-92a expression change [50]. A further possibility is alternative histone modification as Egr2 (induced 4.3-fold after NETD) recruits histone demethylase Jarid1b to the miR-17-92a promotor resulting in histone H3 lysine K4 demethylation and thus repression of cluster transcription [51].

The 3’UTR of CCND1 contains one predicted conserved miRNA binding site for all six miRNAs. We therefore tested the regulative effects of the miR-17 family on the expression of CCND1. All miRNAs except miR-93 were able to repress the activity of reporter genes as well as to reduce CCND1 protein expression. Ottman and colleges showed an impact of miR-17-92a cluster on the expression of CCND1 protein in PC-3 cells though without a proof for direct interaction of cluster miRNAs to the CCND1 3’UTR and discrimination of the miR-17-92a cluster contained miRNAs [52]. We reveal a direct binding of miR-17, -20a, -20b, -106a and -106b to the predicted target site in CCND1 3’UTR by reporter gene assays, additionally confirmed by site directed mutagenesis.

Overexpression of miR-17, -20a, -20b, 106a, and -106b resulted in reduced cell proliferation, colony formation capability and increased apoptosis of LNCaP cells. Members of the analyzed miR-17 family, which share a common seed sequence, as well as the miR-17-92a cluster, which contains miR-17, -18a, -19a/b, -20a and miR-92 family, show a diverse expression pattern in different tumor entities and can either promote or inhibit carcinogenesis. MiR-17-92a cluster and miR-106b have oncogenic roles in many tumor types including HCC, hematopoietic malignancies, medulloblastomas, neuroblastomas, small-cell lung cancer and colon carcinoma. Cai and colleagues could demonstrate mir-17 induction in cervical cancer leading to induced cell proliferation and migration by targeting TGFBR2 [53]. TGFBR2 is induced after NETD and thus could also be responsible for the observed diminished cell proliferation of NE-like LNCaP cells. On the other hand, enforced miR-17 expression reduces proliferation in breast cancer cells, at least in part due to repression of the AIB1 gene [54]. Sun and colleagues could confirm these findings concerning tumor suppressive properties showing an increased cell viability and migration of glioma cells after decrease of miR-17 expression [55]. In prostate tumors and cell lines, the expression pattern as well as the function of this miRNA family is diversely described. Comparable to our results, Ottman and colleges could show a downregulation of miR-17-92a cluster as well as miR-106a in LNCaP cells after CDX treatment and androgen blockade [52]. Furthermore, Hong and colleagues currently described an inhibition of LNCaP cell proliferation as well as an induction of apoptosis after upregulation of miR-17 which supports our results [56]. Interestingly, Gong and colleagues confirm the observed repression of LNCaP cell growth after overexpression of miR-17 [57] in contrary to Yang and colleges showing induced cell proliferation and survival [58]. Due to these conflictive results the function of miR-17 family and miR-17-92a cluster needs further investigation, both in common prostate cancer and during neuroendocrine transdifferentiation.

## Conclusions

We show that androgen deprivation of LNCaP cells leads to dramatic changes in gene and miRNA expression as well as signaling pathway deregulation. The resulting repression of miR-17 family is responsible for up-regulation of CCND1 due to the absence of its post-transcriptional control. Furthermore, the in vitro experiments reveal that increased expression levels of the miR-17 family members suppress cell proliferation and colony forming ability and induce apoptosis. This study gives further hints for tumor suppressor functions of the miR-17 family, whose role in prostate cancer is controversial. In summary, the changes of the mRNA and miRNA expression profile after androgen deprivation will help to find new regulatory mechanisms of NETD in prostate cancer and to identify attractive targets for therapeutic intervention concerning progressive NETD in PCa patients.

## Supporting information

S1 Fig Overexpression of miRNAs after transient transfection with expression plasmids. HEK293T or LNCaP cells were transfected either with control vector or miRNA expression vectors. 48 hours post-transfection total RNA was isolated and miRNA expression was analyzed by qRT-PCR.
(TIF)

S2 Fig Response of TGFB2 3’UTR and protein expression towards miR-148a. The expression of TGFB2 and miR-148a (A) that were assumed to be elevated or reduced according to their signals in microarray (black bars) was assessed by qRT-PCR (grey bars). TGFB2 was predicted to be elevated while miR148a was predicted to be reduced in NE-transdifferentiated LNCaP as compared to untreated cells. *,p<0.05 (B) The TGFB2 3’UTR was cloned behind the luciferase reporter gene of the pMIR vector and the potential binding site for miR-148a in the 3’UTR was additionally mutated by site directed mutagenesis (TGFB2 mut). The reporter gene construct was expressed with the miRNA expression construct or with the empty pSG5 vector as control in the indicated combinations. Results represent the mean of at least 4 independent experiments performed in duplicates. The luciferase activity of the empty luciferase reporter plasmid with the empty pSG5 vector was set to 100%. ***,p<0.001. (C) LNCaP cells were transfected either with control vector or miRNA expression vectors. 48 hours post-transfection the protein expression of TGFB2 was determined by Western blot using ß-actin as loading control. The densitometrical quantification of Western Blots represents the relative downregulation of TGFB2 expression as determined in four independent experiments in relation to the corresponding ß-actin band as loading control.
(TIF)

S3 Fig Original CCND1 and ß-actin blot from Fig 5. (TIF)

S4 Fig Original TGFB2 and ß-actin blot from S2 Fig. (TIF)

S5 Fig Original agarose gels with amplificated RT-PCR fragments from Fig 1. (TIF)

S1 Table Primer sequences. (PDF)

## References

[1] Ferlay J, Soerjomataram I, Dikshit R, Eser S, Mathers C, Rebelo M, et al Cancer incidence and mortality worldwide: sources, methods and major patterns in GLOBOCAN 2012. International journal of cancer. 2015;136(5):E359–86. 10.1002/ijc.29210 . 25220842

[2] Balk SP. Androgen receptor as a target in androgen-independent prostate cancer. Urology. 2002;60(3 Suppl 1):132–8; discussion 8–9. . 12231070 10.1016/s0090-4295(02)01593-5

[3] Perrot V. Neuroendocrine Differentiation in the Progression of Prostate Cancer: An Update on Recent Developments. Open Journal of Urology. 2012;02(03):173–82. 10.4236/oju.2012.223032

[4] Vashchenko N, Abrahamsson PA. Neuroendocrine differentiation in prostate cancer: implications for new treatment modalities. European urology. 2005;47(2):147–55. 10.1016/j.eururo.2004.09.007 . 15661408

[5] Krijnen JL, Janssen PJ, Ruizeveld de Winter JA, van Krimpen H, Schroder FH, van der Kwast TH. Do neuroendocrine cells in human prostate cancer express androgen receptor? Histochemistry. 1993;100(5):393–8. . 8307781 10.1007/BF00268938

[6] Uchida K, Masumori N, Takahashi A, Itoh N, Kato K, Matusik RJ, et al Murine androgen-independent neuroendocrine carcinoma promotes metastasis of human prostate cancer cell line LNCaP. The Prostate. 2006;66(5):536–45. 10.1002/pros.20369 . 16372327

[7] Berruti A, Mosca A, Tucci M, Terrone C, Torta M, Tarabuzzi R, et al Independent prognostic role of circulating chromogranin A in prostate cancer patients with hormone-refractory disease. Endocrine-related cancer. 2005;12(1):109–17. 10.1677/erc.1.00876 . 15788643

[8] Yuan TC, Veeramani S, Lin MF. Neuroendocrine-like prostate cancer cells: neuroendocrine transdifferentiation of prostate adenocarcinoma cells. Endocrine-related cancer. 2007;14(3):531–47. 10.1677/ERC-07-0061 . 17914087

[9] Coppola V, De Maria R, Bonci D. MicroRNAs and prostate cancer. Endocrine-related cancer. 2010;17(1):F1–17. 10.1677/ERC-09-0172 . 19779034

[10] Maugeri-Sacca M, Coppola V, Bonci D, De Maria R. MicroRNAs and prostate cancer: from preclinical research to translational oncology. Cancer journal. 2012;18(3):253–61. 10.1097/PPO.0b013e318258b5b6 . 22647362

[11] Hart M, Nolte E, Wach S, Szczyrba J, Taubert H, Rau TT, et al Comparative microRNA profiling of prostate carcinomas with increasing tumor stage by deep sequencing. Molecular cancer research: MCR. 2014;12(2):250–63. 10.1158/1541-7786.MCR-13-0230 . 24337069

[12] Wach S, Nolte E, Szczyrba J, Stohr R, Hartmann A, Orntoft T, et al MicroRNA profiles of prostate carcinoma detected by multiplatform microRNA screening. International journal of cancer. 2012;130(3):611–21. 10.1002/ijc.26064 . 21400514

[13] Hart M, Wach S, Nolte E, Szczyrba J, Menon R, Taubert H, et al The proto-oncogene ERG is a target of microRNA miR-145 in prostate cancer. The FEBS journal. 2013;280(9):2105–16. 10.1111/febs.12236 . 23480797

[14] Szczyrba J, Nolte E, Hart M, Doll C, Wach S, Taubert H, et al Identification of ZNF217, hnRNP-K, VEGF-A and IPO7 as targets for microRNAs that are downregulated in prostate carcinoma. International journal of cancer. 2013;132(4):775–84. 10.1002/ijc.27731 . 22815235

[15] Szczyrba J, Nolte E, Wach S, Kremmer E, Stohr R, Hartmann A, et al Downregulation of Sec23A protein by miRNA-375 in prostate carcinoma. Molecular cancer research: MCR. 2011;9(6):791–800. 10.1158/1541-7786.MCR-10-0573 . 21593139

[16] Ding M, Lin B, Li T, Liu Y, Li Y, Zhou X, et al A dual yet opposite growth-regulating function of miR-204 and its target XRN1 in prostate adenocarcinoma cells and neuroendocrine-like prostate cancer cells. Oncotarget. 2015;6(10):7686–700. 10.18632/oncotarget.3480 ; PubMed Central PMCID: PMC4480709. 25797256 PMC4480709

[17] Zheng C, Yinghao S, Li J. MiR-221 expression affects invasion potential of human prostate carcinoma cell lines by targeting DVL2. Medical oncology. 2012;29(2):815–22. 10.1007/s12032-011-9934-8 . 21487968

[18] Limbert C, Ebert R, Schilling T, Path G, Benisch P, Klein-Hitpass L, et al Functional signature of human islet-derived precursor cells compared to bone marrow-derived mesenchymal stem cells. Stem cells and development. 2010;19(5):679–91. 10.1089/scd.2009.0241 . 19895235

[19] Tusher VG, Tibshirani R, Chu G. Significance analysis of microarrays applied to the ionizing radiation response. Proceedings of the National Academy of Sciences of the United States of America. 2001;98(9):5116–21. 10.1073/pnas.091062498 ; PubMed Central PMCID: PMC33173. 11309499 PMC33173

[20] Imig J, Motsch N, Zhu JY, Barth S, Okoniewski M, Reineke T, et al microRNA profiling in Epstein-Barr virus-associated B-cell lymphoma. Nucleic acids research. 2011;39(5):1880–93. 10.1093/nar/gkq1043 ; PubMed Central PMCID: PMC3061055. 21062812 PMC3061055

[21] Huang da W, Sherman BT, Lempicki RA. Bioinformatics enrichment tools: paths toward the comprehensive functional analysis of large gene lists. Nucleic acids research. 2009;37(1):1–13. Epub 2008/11/27. 10.1093/nar/gkn923 ; PubMed Central PMCID: PMCPMC2615629. 19033363 PMC2615629

[22] Huang da W, Sherman BT, Lempicki RA. Systematic and integrative analysis of large gene lists using DAVID bioinformatics resources. Nature protocols. 2009;4(1):44–57. Epub 2009/01/10. 10.1038/nprot.2008.211 . 19131956

[23] Fukami K, Sekiguchi F, Yasukawa M, Asano E, Kasamatsu R, Ueda M, et al Functional upregulation of the H2S/Cav3.2 channel pathway accelerates secretory function in neuroendocrine-differentiated human prostate cancer cells. Biochemical pharmacology. 2015;97(3):300–9. 10.1016/j.bcp.2015.08.005 . 26256074

[24] Hulsegge I, Kommadath A, Smits MA. Globaltest and GOEAST: two different approaches for Gene Ontology analysis. BMC proceedings. 2009;3 Suppl 4:S10 10.1186/1753-6561-3-S4-S10 ; PubMed Central PMCID: PMC2712740. 19615110 PMC2712740

[25] Zhang W, Li Y. miR-148a downregulates the expression of transforming growth factor-beta2 and SMAD2 in gastric cancer. International journal of oncology. 2016;48(5):1877–85. 10.3892/ijo.2016.3437 ; PubMed Central PMCID: PMC4809655. 26983401 PMC4809655

[26] Shen R, Dorai T, Szaboles M, Katz AE, Olsson CA, Buttyan R. Transdifferentiation of cultured human prostate cancer cells to a neuroendocrine cell phenotype in a hormone-depleted medium. Urologic oncology. 1997;3(2):67–75. . 21227062 10.1016/s1078-1439(97)00039-2

[27] Cerasuolo M, Paris D, Iannotti FA, Melck D, Verde R, Mazzarella E, et al Neuroendocrine Transdifferentiation in Human Prostate Cancer Cells: An Integrated Approach. Cancer research. 2015;75(15):2975–86. Epub 2015/06/13. 10.1158/0008-5472.CAN-14-3830 . 26069250

[28] Martin-Orozco RM, Almaraz-Pro C, Rodriguez-Ubreva FJ, Cortes MA, Ropero S, Colomer R, et al EGF prevents the neuroendocrine differentiation of LNCaP cells induced by serum deprivation: the modulator role of PI3K/Akt. Neoplasia. 2007;9(8):614–24. ; PubMed Central PMCID: PMC1950431. 17898861 10.1593/neo.07337 PMC1950431

[29] Zelivianski S, Verni M, Moore C, Kondrikov D, Taylor R, Lin MF. Multipathways for transdifferentiation of human prostate cancer cells into neuroendocrine-like phenotype. Biochimica et biophysica acta. 2001;1539(1–2):28–43. Epub 2001/06/08. . 11389966 10.1016/s0167-4889(01)00087-8

[30] Deeble PD, Murphy DJ, Parsons SJ, Cox ME. Interleukin-6- and cyclic AMP-mediated signaling potentiates neuroendocrine differentiation of LNCaP prostate tumor cells. Molecular and cellular biology. 2001;21(24):8471–82. 10.1128/MCB.21.24.8471-8482.2001 ; PubMed Central PMCID: PMC100010. 11713282 PMC100010

[31] Bieberich CJ, Fujita K, He WW, Jay G. Prostate-specific and androgen-dependent expression of a novel homeobox gene. The Journal of biological chemistry. 1996;271(50):31779–82. . 8943214 10.1074/jbc.271.50.31779

[32] Lin B, Ferguson C, White JT, Wang S, Vessella R, True LD, et al Prostate-localized and androgen-regulated expression of the membrane-bound serine protease TMPRSS2. Cancer research. 1999;59(17):4180–4. . 10485450

[33] Ma AH, Xia L, Desai SJ, Boucher DL, Guan Y, Shih HM, et al Male germ cell-associated kinase, a male-specific kinase regulated by androgen, is a coactivator of androgen receptor in prostate cancer cells. Cancer research. 2006;66(17):8439–47. 10.1158/0008-5472.CAN-06-1636 . 16951154

[34] Hsieh T, Wu J. Differential expression and regulation of p53 in human prostatic cells. International journal of oncology. 1997;10(6):1109–12. . 21533491

[35] Czapiewski P, Bobowicz M, Peksa R, Skrzypski M, Gorczynski A, Szczepanska-Michalska K, et al Keratin 7 expression in lymph node metastases but not in the primary tumour correlates with distant metastases and poor prognosis in colon carcinoma. Polish journal of pathology: official journal of the Polish Society of Pathologists. 2016;67(3):228–34. 10.5114/pjp.2016.63774 . 28155971

[36] Eder IE, Haag P, Basik M, Mousses S, Bektic J, Bartsch G, et al Gene expression changes following androgen receptor elimination in LNCaP prostate cancer cells. Molecular carcinogenesis. 2003;37(4):181–91. 10.1002/mc.10136 . 12891627

[37] Terry S, Maille P, Baaddi H, Kheuang L, Soyeux P, Nicolaiew N, et al Cross modulation between the androgen receptor axis and protocadherin-PC in mediating neuroendocrine transdifferentiation and therapeutic resistance of prostate cancer. Neoplasia. 2013;15(7):761–72. ; PubMed Central PMCID: PMC3689239. 23814488 10.1593/neo.122070 PMC3689239

[38] Pashaei E, Pashaei E, Ahmady M, Ozen M, Aydin N. Meta-analysis of miRNA expression profiles for prostate cancer recurrence following radical prostatectomy. PloS one. 2017;12(6):e0179543 10.1371/journal.pone.0179543 . 28651018 PMC5484492

[39] Das SG, Romagnoli M, Mineva ND, Barille-Nion S, Jezequel P, Campone M, et al miR-720 is a downstream target of an ADAM8-induced ERK signaling cascade that promotes the migratory and invasive phenotype of triple-negative breast cancer cells. Breast cancer research: BCR. 2016;18(1):40 10.1186/s13058-016-0699-z ; PubMed Central PMCID: PMC4818899. 27039296 PMC4818899

[40] Torres S, Garcia-Palmero I, Bartolome RA, Fernandez-Acenero MJ, Molina E, Calvino E, et al Combined miRNA profiling and proteomics demonstrates that different miRNAs target a common set of proteins to promote colorectal cancer metastasis. The Journal of pathology. 2017;242(1):39–51. 10.1002/path.4874 . 28054337

[41] Tang Y, Lin Y, Li C, Hu X, Liu Y, He M, et al MicroRNA-720 promotes in vitro cell migration by targeting Rab35 expression in cervical cancer cells. Cell & bioscience. 2015;5:56 10.1186/s13578-015-0047-5 ; PubMed Central PMCID: PMC4583841. 26413265 PMC4583841

[42] Sun X, Liu Z, Yang Z, Xiao L, Wang F, He Y, et al Association of microRNA-126 expression with clinicopathological features and the risk of biochemical recurrence in prostate cancer patients undergoing radical prostatectomy. Diagnostic pathology. 2013;8:208 10.1186/1746-1596-8-208 ; PubMed Central PMCID: PMC3928806. 24350576 PMC3928806

[43] Murata T, Takayama K, Katayama S, Urano T, Horie-Inoue K, Ikeda K, et al miR-148a is an androgen-responsive microRNA that promotes LNCaP prostate cell growth by repressing its target CAND1 expression. Prostate cancer and prostatic diseases. 2010;13(4):356–61. 10.1038/pcan.2010.32 . 20820187

[44] Szczyrba J, Loprich E, Wach S, Jung V, Unteregger G, Barth S, et al The microRNA profile of prostate carcinoma obtained by deep sequencing. Molecular cancer research: MCR. 2010;8(4):529–38. 10.1158/1541-7786.MCR-09-0443 . 20353999

[45] Waltering KK, Porkka KP, Jalava SE, Urbanucci A, Kohonen PJ, Latonen LM, et al Androgen regulation of micro-RNAs in prostate cancer. The Prostate. 2011;71(6):604–14. 10.1002/pros.21276 . 20945501

[46] Viticchie G, Lena AM, Latina A, Formosa A, Gregersen LH, Lund AH, et al MiR-203 controls proliferation, migration and invasive potential of prostate cancer cell lines. Cell cycle. 2011;10(7):1121–31. 10.4161/cc.10.7.15180 . 21368580

[47] Saini S, Majid S, Yamamura S, Tabatabai L, Suh SO, Shahryari V, et al Regulatory Role of mir-203 in Prostate Cancer Progression and Metastasis. Clinical cancer research: an official journal of the American Association for Cancer Research. 2011;17(16):5287–98. 10.1158/1078-0432.CCR-10-2619 . 21159887

[48] Schiewer MJ, Morey LM, Burd CJ, Liu Y, Merry DE, Ho SM, et al Cyclin D1 repressor domain mediates proliferation and survival in prostate cancer. Oncogene. 2009;28(7):1016–27. 10.1038/onc.2008.446 ; PubMed Central PMCID: PMC2852245. 19079343 PMC2852245

[49] Guo J, Mei Y, Li K, Huang X, Yang H. Downregulation of miR-17-92a cluster promotes autophagy induction in response to celastrol treatment in prostate cancer cells. Biochemical and biophysical research communications. 2016;478(2):804–10. 10.1016/j.bbrc.2016.08.029 . 27501757

[50] O'Donnell KA, Wentzel EA, Zeller KI, Dang CV, Mendell JT. c-Myc-regulated microRNAs modulate E2F1 expression. Nature. 2005;435(7043):839–43. 10.1038/nature03677 . 15944709

[51] Pospisil V, Vargova K, Kokavec J, Rybarova J, Savvulidi F, Jonasova A, et al Epigenetic silencing of the oncogenic miR-17-92 cluster during PU.1-directed macrophage differentiation. The EMBO journal. 2011;30(21):4450–64. 10.1038/emboj.2011.317 ; PubMed Central PMCID: PMC3230374. 21897363 PMC3230374

[52] Ottman R, Levy J, Grizzle WE, Chakrabarti R. The other face of miR-17-92a cluster, exhibiting tumor suppressor effects in prostate cancer. Oncotarget. 2016;7(45):73739–53. 10.18632/oncotarget.12061 ; PubMed Central PMCID: PMC5340125. 27650539 PMC5340125

[53] Cai N, Hu L, Xie Y, Gao JH, Zhai W, Wang L, et al MiR-17-5p promotes cervical cancer cell proliferation and metastasis by targeting transforming growth factor-beta receptor 2. European review for medical and pharmacological sciences. 2018;22(7):1899–906. Epub 2018/04/25. 10.26355/eurrev_201804_14712 . 29687841

[54] Hossain A, Kuo MT, Saunders GF. Mir-17-5p regulates breast cancer cell proliferation by inhibiting translation of AIB1 mRNA. Molecular and cellular biology. 2006;26(21):8191–201. 10.1128/MCB.00242-06 ; PubMed Central PMCID: PMC1636750. 16940181 PMC1636750

[55] Sun G, SiMa G, Wu C, Fan Y, Tan Y, Wang Z, et al Decreased MiR-17 in glioma cells increased cell viability and migration by increasing the expression of Cyclin D1, p-Akt and Akt. PloS one. 2018;13(1):e0190515 Epub 2018/01/20. 10.1371/journal.pone.0190515 ; PubMed Central PMCID: PMCPMC5774692. 29351283 PMC5774692

[56] Dai H, Wang C, Yu Z, He D, Yu K, Liu Y, et al MiR-17 Regulates Prostate Cancer Cell Proliferation and Apoptosis Through Inhibiting JAK-STAT3 Signaling Pathway. Cancer biotherapy & radiopharmaceuticals. 2018;33(3):103–9. Epub 2018/04/12. 10.1089/cbr.2017.2386 . 29641255

[57] Gong AY, Eischeid AN, Xiao J, Zhao J, Chen D, Wang ZY, et al miR-17-5p targets the p300/CBP-associated factor and modulates androgen receptor transcriptional activity in cultured prostate cancer cells. BMC cancer. 2012;12:492 10.1186/1471-2407-12-492 ; PubMed Central PMCID: PMC3519561. 23095762 PMC3519561

[58] Yang X, Du WW, Li H, Liu F, Khorshidi A, Rutnam ZJ, et al Both mature miR-17-5p and passenger strand miR-17-3p target TIMP3 and induce prostate tumor growth and invasion. Nucleic acids research. 2013;41(21):9688–704. 10.1093/nar/gkt680 ; PubMed Central PMCID: PMC3834805. 23990326 PMC3834805

