# Supplementary figures and images for: The deregulation of miR-17/CCND1 axis during neuroendocrine transdifferentiation of LNCaP prostate cancer cells
Source: PLoS One. 2018 Jul 12;13(7):e0200472. doi: 10.1371/journal.pone.0200472 (PMC6042731; doi:10.1371/journal.pone.0200472)

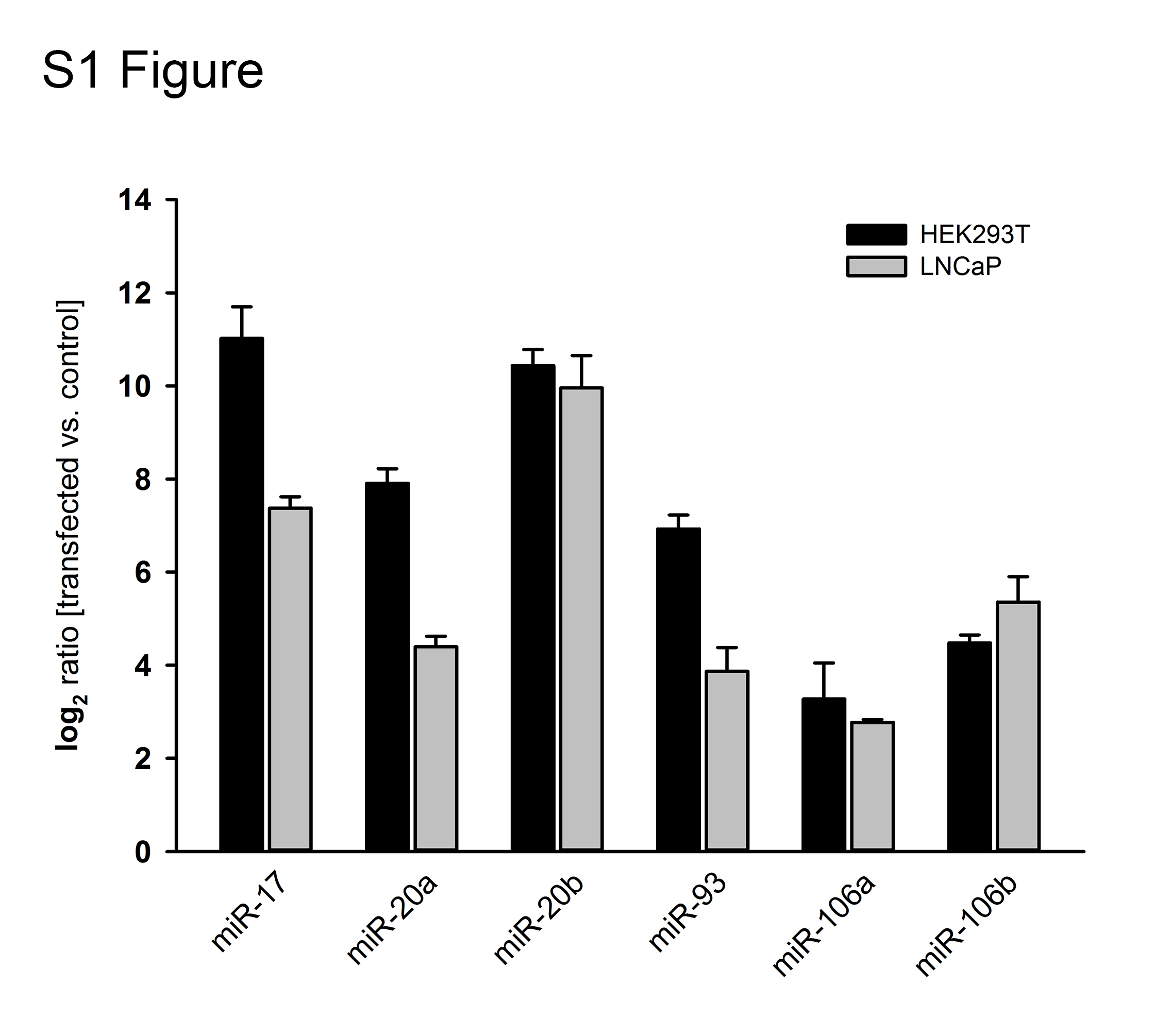

Supplement: S1 Fig — HEK293T or LNCaP cells were transfected either with control vector or miRNA expression vectors. 48 hours post-transfection total RNA was isolated and miRNA expression was analyzed by qRT-PCR. (TIF) [file pone.0200472.s001.tif]

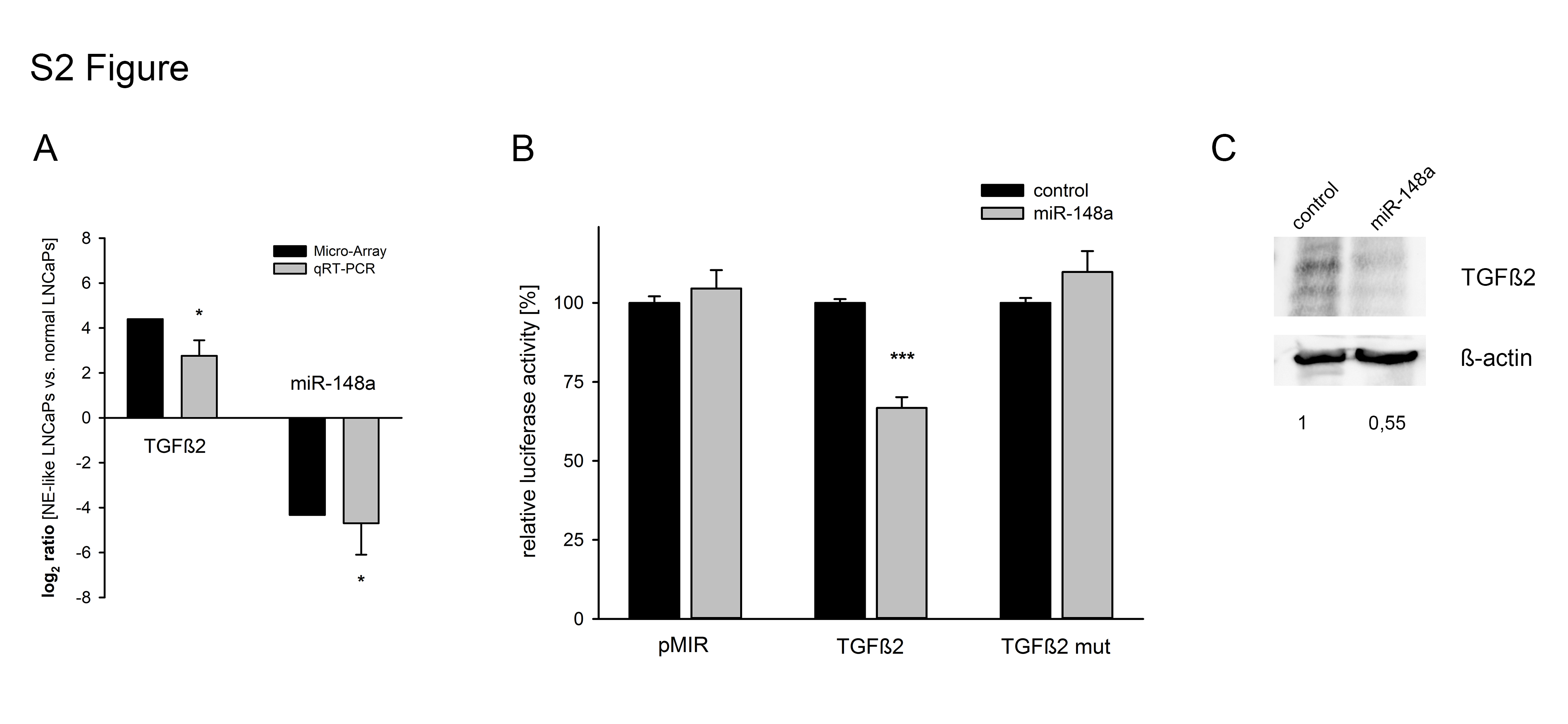

Supplement: S2 Fig — The expression of TGFB2 and miR-148a (A) that were assumed to be elevated or reduced according to their signals in microarray (black bars) was assessed by qRT-PCR (grey bars). TGFB2 was predicted to be elevated while miR148a was predicted to be reduced in NE-transdifferentiated LNCaP as compared to untreated cells. *,p<0.05 (B) The TGFB2 3’UTR was cloned behind the luciferase reporter gene of the pMIR vector and the potential binding site for miR-148a in the 3’UTR was additionally mutated by site directed mutagenesis (TGFB2 mut). The reporter gene construct was expressed with the miRNA expression construct or with the empty pSG5 vector as control in the indicated combinations. Results represent the mean of at least 4 independent experiments performed in duplicates. The luciferase activity of the empty luciferase reporter plasmid with the empty pSG5 vector was set to 100%. ***,p<0.001. (C) LNCaP cells were transfected either with control vector or miRNA expression vectors. 48 hours post-transfection the protein expression of TGFB2 was determined by Western blot using ß-actin as loading control. The densitometrical quantification of Western Blots represents the relative downregulation of TGFB2 expression as determined in four independent experiments in relation to the corresponding ß-actin band as loading control. (TIF) [file pone.0200472.s002.tif]

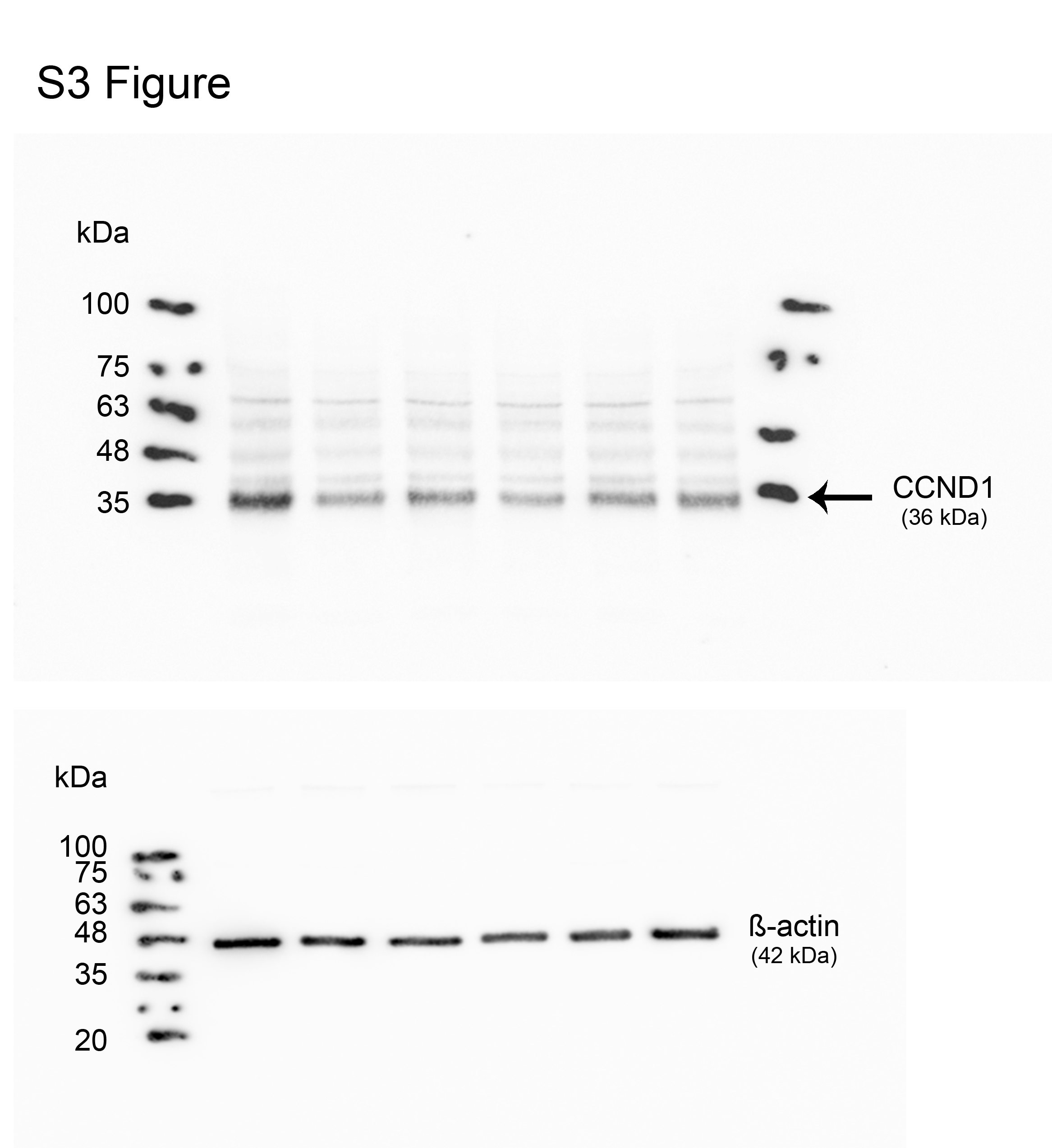

Supplement: S3 Fig — (TIF) [file pone.0200472.s003.tif]

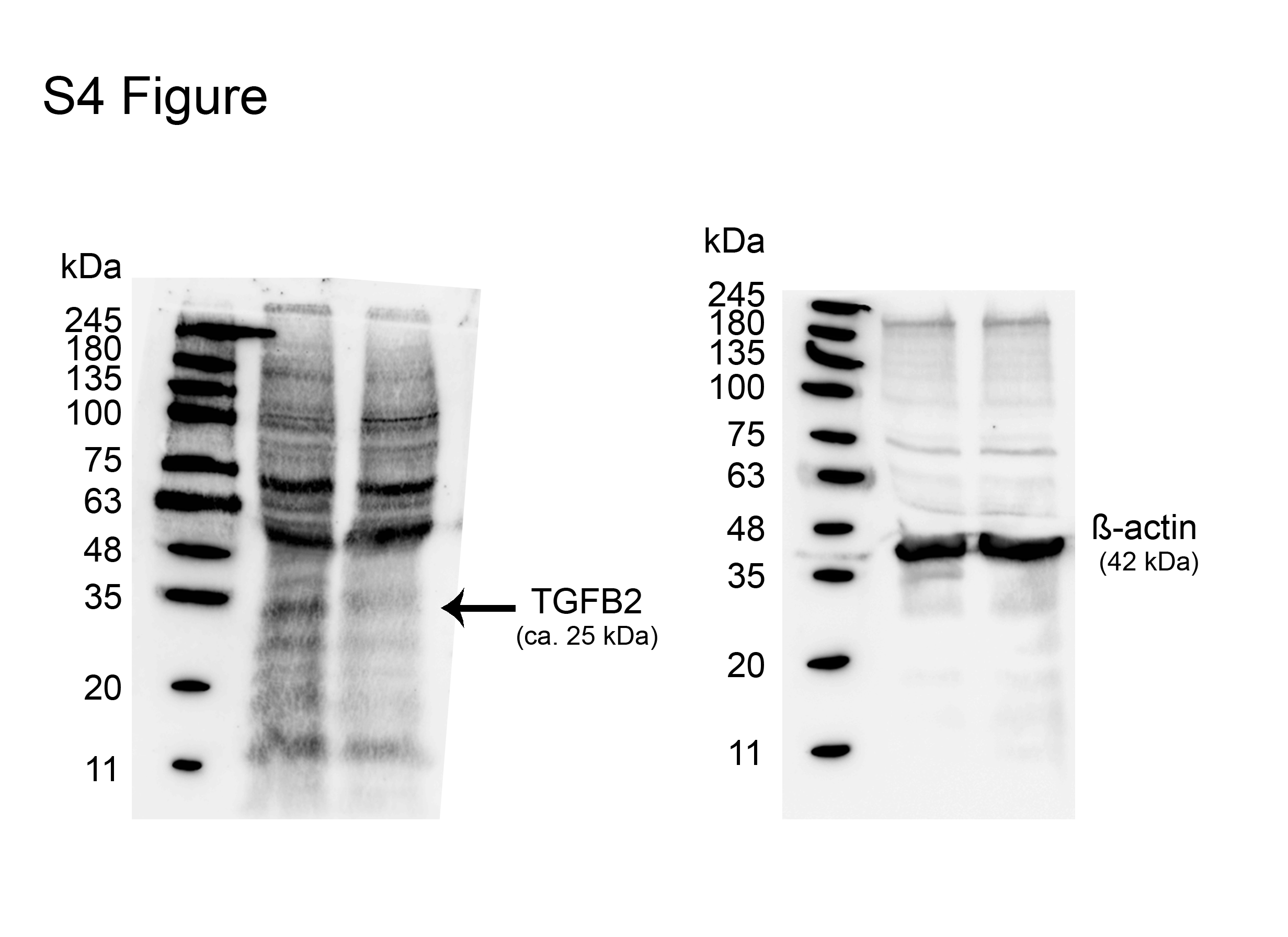

Supplement: S4 Fig — (TIF) [file pone.0200472.s004.tif]

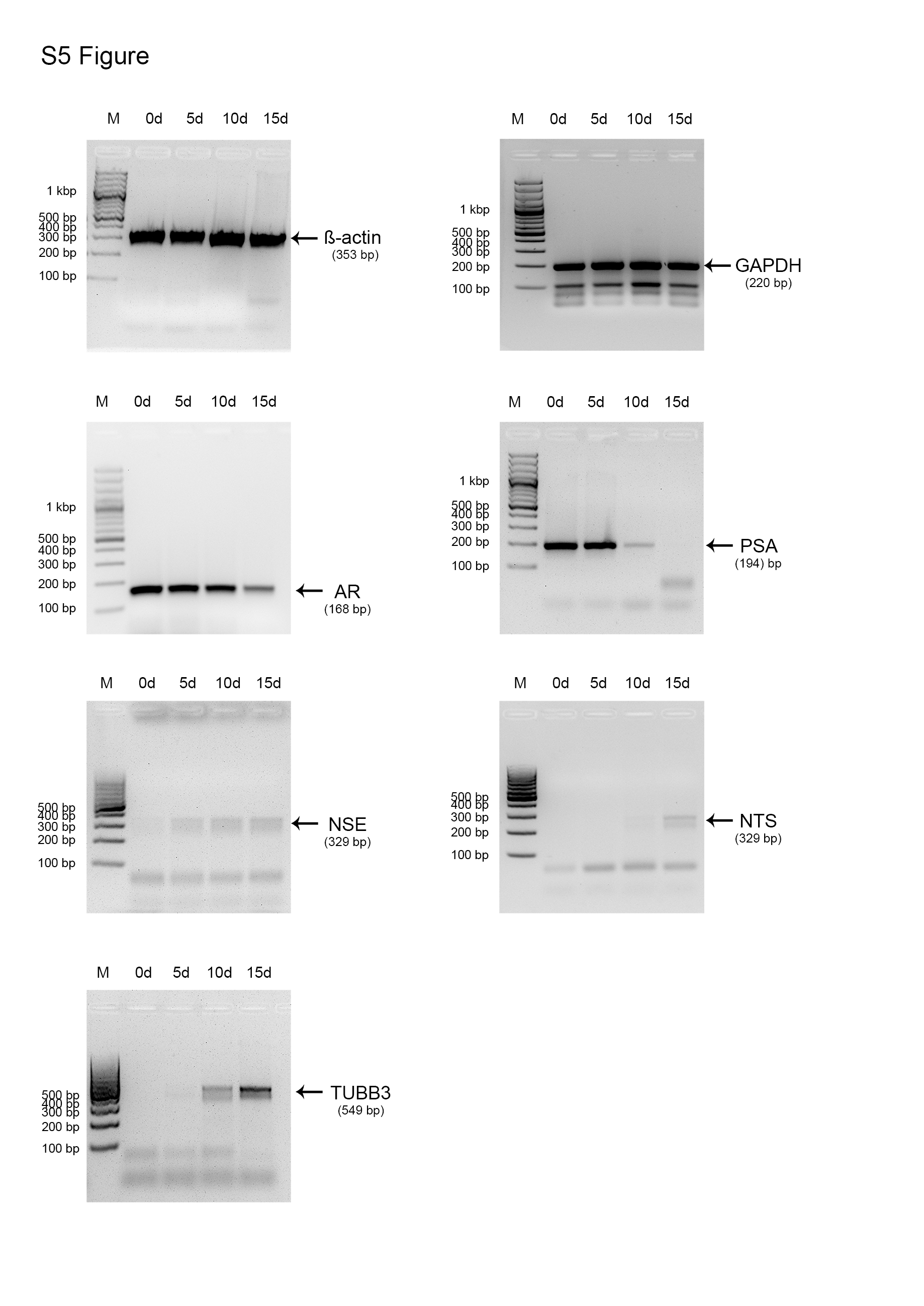

Supplement: S5 Fig — (TIF) [file pone.0200472.s005.tif]
