# Supplementary material for: The deregulation of miR-17/CCND1 axis during neuroendocrine transdifferentiation of LNCaP prostate cancer cells
Source: PLoS One. 2018 Jul 12;13(7):e0200472. doi: 10.1371/journal.pone.0200472 (PMC6042731; doi:10.1371/journal.pone.0200472)
Supplement: S1 Table — (PDF) [file pone.0200472.s006.pdf]

S1 Table: Primer sequences

**RT-PCR**	
AR-for	5'-cctggcttccgcaacttacac-3'
AR-rev	5'-ggacttgtgcatgcggtactc-3'
Beta-actin-for	5'-gctcgtcgtcgacaacggctc-3'
Beta-actin-rev	5'-caaacatgatctgggtcatcttctc-3'
GAPDH-for	5'-gatttggtcgtattggg-3'
GAPDH-rev	5'-tccacgacgtactcagc-3'
NSE-for	5'-tctgcagtcccgagatcccagc-3'
NSE-rev	5'-ctgatgagggctggcgcgat-3'
NTS-for	5'-catgctactcctggctttcagct-3'
NTS-rev	5'-actcccagtgttgaaaagccct-3'
PSA-for	5'-tccaatgacgtgtgtgcgca-3'
PSA-rev	5'-gtgtacagggaaggcctttc-3'
TUBB3-for	5'-ctcgagccattctggtggacc-3'
TUBB3-rev	5'-gatgtcgtagagcgcctcgtt-3'
**qRT-PCR**	
ADAM7-for	5'-cagtgcaatgaaaatcctgtg-3'
ADAM7-rev	5'-tacaggtgcctgtccttcct-3'
CCND1-for	5'-gctgtgcatctacacccgaca-3'
CCND1-rev	5'-ttgagcttgttcaccaggag-3'
CNKSR2-for	5'-tggcgatgaagtgattcaag-3'
CNKSR2-rev	5'-actcgggtcctctcgtagtg-3'
CPEB1-for	5'-agagcagcacacactcggta-3'
CPEB1-rev	5'-agaggcaggaagctcaagg-3'
KCNN2-for	5'-gccttatcagtctctccacga-3'
KCNN2-rev	5'-ctgctccattgtccacca-3'
KLK2-for	5'-ctgcccattgcctaaagaag-3'
KLK2-rev	5'-accctctggcctgtgtctt-3'
KRT34-for	5'-ggagagtgaggactgcaagc-3'
KRT34-rev	5'-ccacaggagttgccactagc-3'
TGM2-for	5'-ggcaccaagtacctgctca-3'
TGM2-rev	5'-agaggatgcaaagaggaacg-3'
TGFB2-for	5'-ccttcttcccctccgaaac-3'
TGFB2-rev	5'-agagcacctgggactgtctg-3'
miR-17-for	5'-caaagtgcttacagtgcaggtag-3'
miR-20a-for	5'-taaagtgcttatagtgcaggtag-3'
miR-20b-for	5'-caaagtgctcatagtgcaggtag-3'
miR-93-for	5'-caaagtgctgttcgtgcaggtag-3'
miR-106a-for	5'-aaaagtgcttacagtgcaggtag-3'
miR-106b-for	5'-taaagtgctgacagtgcagat-3'
miR-126-for	5'-tcgtaccgtgagtaataatgcg-3'
miR-141-for	5'-taacactgtctggtaaagatgg-3'
miR-148a-for	5'-tcagtgcactacagaactttgt-3'
miR-203-for	5'-gtgaaatgtttaggaccactag-3'
miR-720-for	5'-tctcgctggggcctcca-3'
miR-1280	5'-tcccaccgctgccaccc-3'
miR-3135b-for	5'-ggctggagcgagtgcagtggtg-3'
miR-3178	5'-ggggcgcggccggatcg-3'

miR-xxx-rev	10x miScript Universal Primer (Qiagen)
**cloning primers**	
CCND1-for-BcuI	5'-ggactagtctgctggttttctacccaacg-3'
CCND1-rev-SacI	5'-cgagctcgccaaaccgtgcctggaagtc-3'
miR-17-for-BamHI	5'-cgggatccagagccaccacttccagtgc-3'
miR-17-rev-BglII	5'-cgagatctactcaacatcagcaggccct-3'
miR-20a-for-EcoRI	5'-cggaattctgcaaaactgatggtggcct-3'
miR-20a-rev-BamHI	5'-cgggatccccacagtcagttttgcatg-3'
miR-20b-for-EcoRI	5'-cggaattcctaaatgccccttctggcacag-3'
miR-20b-rev-BamHI	5'-cgggatcctgcatggatttgcacagcca-3'
miR-106a-for-EcoRI	5'-cggaattcgcttagactctgtaagccaa-3'
miR-106a-rev-BamHI	5'-cgggatcctacgctgaaatgcaaacctg-3'
miR-106b-for-EcoRI	5'-cggaattctggtaagtgcccaaattgct-3'
miR-106b-for-BamHI	5'-cgggatccagcacaggatctaggacaca-3'
miR-93-for-EcoRI	5’-cggaattcggggtagctccttaccgtgc-3’
miR-93-rev-BamHI	5’- cgggatccacaggagtggaatccccct-3’
**site directed mutagenesis**	
CCND1-mut-for	5'-ccattccatttccaaCGTCGACcagtccaataggtgt-3'
CCND1-mut-rev	5'-acacctattggactgGTCGACGttggaaatggaatgg-3'
